# screenwerk: a modular tool for the design and analysis of drug combination screens

**DOI:** 10.1093/bioinformatics/btac840

**Published:** 2022-12-27

**Authors:** Robert Hanes, Pilar Ayuda-Durán, Leiv Rønneberg, Sigve Nakken, Eivind Hovig, Manuela Zucknick, Jorrit M Enserink

**Affiliations:** Department of Molecular Cell Biology, Institute for Cancer Research, Oslo University Hospital, 0379 Oslo, Norway; Centre for Cancer Cell Reprogramming, Faculty of Medicine, Institute of Clinical Medicine, University of Oslo, 0318 Oslo, Norway; Section for Biochemistry and Molecular Biology, Faculty of Mathematics and Natural Sciences, University of Oslo, 0316 Oslo, Norway; Department of Molecular Cell Biology, Institute for Cancer Research, Oslo University Hospital, 0379 Oslo, Norway; Centre for Cancer Cell Reprogramming, Faculty of Medicine, Institute of Clinical Medicine, University of Oslo, 0318 Oslo, Norway; Oslo Centre for Biostatistics and Epidemiology (OCBE), University of Oslo, 0317 Oslo, Norway; MRC Biostatistics Unit, University of Cambridge, Cambridge CB2 0SR, UK; Centre for Cancer Cell Reprogramming, Faculty of Medicine, Institute of Clinical Medicine, University of Oslo, 0318 Oslo, Norway; Department of Tumor Biology, Institute for Cancer Research, Oslo University Hospital, Oslo 0379, Norway; Department of Informatics, Centre for Bioinformatics, University of Oslo, Oslo 0372, Norway; Department of Tumor Biology, Institute for Cancer Research, Oslo University Hospital, Oslo 0379, Norway; Department of Informatics, Centre for Bioinformatics, University of Oslo, Oslo 0372, Norway; Oslo Centre for Biostatistics and Epidemiology (OCBE), University of Oslo, 0317 Oslo, Norway; Department of Molecular Cell Biology, Institute for Cancer Research, Oslo University Hospital, 0379 Oslo, Norway; Centre for Cancer Cell Reprogramming, Faculty of Medicine, Institute of Clinical Medicine, University of Oslo, 0318 Oslo, Norway; Section for Biochemistry and Molecular Biology, Faculty of Mathematics and Natural Sciences, University of Oslo, 0316 Oslo, Norway

## Abstract

**Motivation:**

There is a rapidly growing interest in high-throughput drug combination screening to identify synergizing drug interactions for treatment of various maladies, such as cancer and infectious disease. This creates the need for pipelines that can be used to design such screens, perform quality control on the data and generate data files that can be analyzed by synergy-finding bioinformatics applications.

**Results:**

screenwerk is an open-source, end-to-end modular tool available as an R-package for the design and analysis of drug combination screens. The tool allows for a customized build of pipelines through its modularity and provides a flexible approach to quality control and data analysis. screenwerk is adaptable to various experimental requirements with an emphasis on precision medicine. It can be coupled to other R packages, such as bayesynergy, to identify synergistic and antagonistic drug interactions in cell lines or patient samples. screenwerk is scalable and provides a complete solution for setting up drug sensitivity screens, read raw measurements and consolidate different datasets, perform various types of quality control and analyze, report and visualize the results of drug sensitivity screens.

**Availability and implementation:**

The R-package and technical documentation is available at https://github.com/Enserink-lab/screenwerk; the R source code is publicly available at https://github.com/Enserink-lab/screenwerk under GNU General Public License v3.0; bayesynergy is accessible at https://github.com/ocbe-uio/bayesynergy. Selected modules are available through Galaxy, an open-source platform for FAIR data analysis at https://oncotools.elixir.no

**Supplementary information:**

Supplementary data are available at *Bioinformatics* online.

## 1 Introduction

The advent of targeted therapy has revolutionized the treatment of many types of cancer. However, despite often eliciting a strong initial response, most targeted therapies ultimately fail due to a variety of reasons, including mutations in the molecular target, overexpression of the drug target or activation of compensatory mechanisms (Bell and Gilan, 2020; Bergholz and Zhao, 2021; Logue and Morrison, 2012). One solution to this problem is the use of combinations of drugs (Bayat Mokhtari *et al.*, 2017; Plana *et al.*, 2022; Saputra *et al.*, 2018). This is exemplified by clinical trials with melanoma, which is a form of cancer frequently driven by mutations that activate the Ras-Raf-MEK-ERK pathway, such as BRAF V600 mutations (Hodis *et al.*, 2012). Ras pathway-driven forms of melanoma can be treated with various kinase inhibitors, including the Raf inhibitors vemurafinib and dabrafenib and the MEK inhibitors trametinib and cobimetinib (Luke and Hodi, 2013; Robert *et al.*, 2015). Randomized phase III clinical trials have demonstrated that Raf inhibitors are associated with increased progression-free survival (Chapman *et al.*, 2011; Hauschild *et al.*, 2012). However, acquired resistance to these single-drug treatments is a major problem, and only a minority of patients showed durable responses (Chapman *et al.*, 2011; Hauschild *et al.*, 2012). Resistance to BRAF inhibitors can occur via multiple mechanisms, although reactivation of the MAPK pathway is a common theme (Solit and Rosen, 2011), which can be partially overcome by combining BRAF inhibitors with MEK inhibitors (Flaherty *et al.*, 2012). Additional combinations of targeted therapy and, more recently, with immunotherapy have been identified that can overcome resistance (Luke *et al.*, 2017). Similar effects have been observed for a wide variety of cancers, including acute myeloid leukemia (AML), lung cancer and breast cancer (Fisusi and Akala, 2019; Latif *et al.*, 2021; Yuan *et al.*, 2019), and combination treatment clearly has the potential to significantly improve survival rates for cancer patients. However, given the sheer number of targeted therapies, identifying synergistic drug combinations is a major challenge, and several obstacles still need to be overcome.

One such obstacle is the lack of an integrated bioinformatics workflow that integrates the design and execution of drug combination screens. Such a workflow should also include quality control measures to identify and reduce technical variability, which is a persistent problem that limits the reproducibility of high-throughput drug screens (Hatzis *et al.*, 2014; Larsson *et al.*, 2020; Niepel *et al.*, 2019). Lack of reproducibility is a well-documented problem in the screening of new drugs for the treatment of cancer. However, including strict quality control measures during the early stages of preclinical development can contribute to reducing the high attrition rates associated with cancer drug sensitivity screens (Larsson *et al.*, 2020). The bioinformatics workflow should also include a data output step that produces data files that can be visualized and analyzed across multiple platforms.

Here, we present screenwerk, which provides a comprehensive end-to-end solution that integrates design, execution, quality control and data analysis of large-scale drug combination screens.

## 2 Materials and methods

### 2.1 Generating drug combination files

**Fig. 1.**
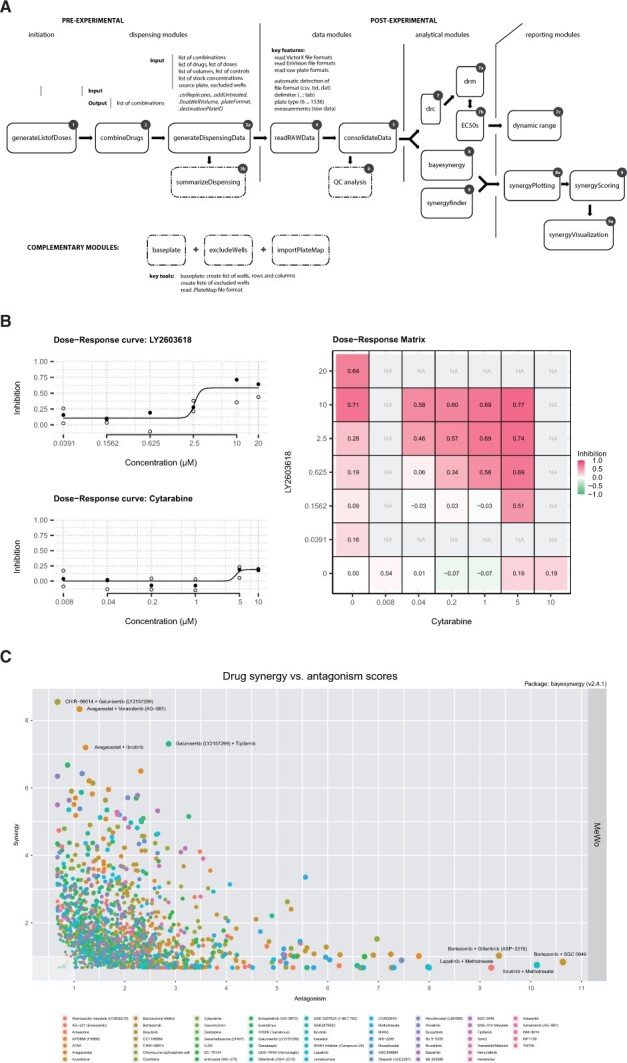
screenwerk overview. **(A**) The modular architecture of screenwerk. (**B**) Dose–response curves and matrix for a drug combination. Left, Single-drug dose response curves for LY2603618 and Cytarabine with measurement points in triplicates for each concentration. Black point indicates the median. Right, Drug response matrix for the combination of these two drugs. Numbers in the matrix indicate the relative reduction in viable cells (as determined by CellTiter-Glo) compared to DMSO-treated cells. (**C**) Plot showing drug synergy versus drug antagonism, as determined by synergy scores (calculated with bayesynergy). The identity of the most synergistic (top left) and most antagonistic (bottom right) drug pairs are indicated in the graph

Screenwerk is a modular software tool to facilitate drug combination screening (Fig. 1A). It takes a list of drugs and creates a drug combination output file that can be used by a pipetting robot to generate a source plate that can subsequently be used to prepare a set of multi-well plates containing drug combinations of interest. This inventory list of drug names and concentrations has to be provided in the form of a comma-separated values file. If desired, screenwerk can generate a visual map of the source plate and target plates (Supplementary Figs S1 and S2). The set of plates containing the drug combinations is then used to screen cells using the desired experimental read-out, such as widely used luminescent cell viability assays (e.g. CellTiter-Glo^®^). Upon completing the drug combination screen, raw data files from various sources can be used by screenwerk to integrate the raw measurements with the original drug combination map, thereby creating a master dataset that now contains important information for downstream analysis, such as plate numbers, bar codes and drug identifiers.

### 2.2 Quality control

High-throughput assays can suffer from technical errors, such as evaporation at the borders of the plate and ‘line effects’ stemming from errors during drug or cell dispension. Therefore, screenwerk performs quality control on the master dataset and generates several plots and heatmaps that provide quantitative and qualitative insight in the overall variance and noise in the experimental controls for each of the multi-well plates in the screen (Supplementary Figs S3–S7). The Z’-factor (Zhang *et al.*, 1999) is also calculated to obtain an overview of the statistical effect size for each of the plates (Supplementary Fig. S8). These visualizations can help the experimenter with assessing the quality of the screen, which is useful not only for identifying and correcting potential technical errors but also for deciding whether to continue with comprehensive downstream analysis of the dataset.

### 2.3 Curve fitting and visualization of drug interactions

Upon completing quality control analysis, screenwerk normalizes the raw measurements to the positive and negative controls and creates files for single-drug responses and for drug combination responses. These single-drug response files are then used for curve fitting using a four-parameter log-logistic function (Vølund, 1978) (Supplementary Figs S9–S12), which is required for calculating EC10, EC50 and EC90 values. Screenwerk also includes a tool to visually identify drug responses that fall outside the dynamic range (Supplementary Figs S13–S15). In addition to calculating single-drug responses, screenwerk generates dose–response matrices to visualize drug-drug interactions (Fig. 1B).

### 2.4 Drug synergy and antagonism

Several tools can be used to identify positive and negative drug–drug interactions (synergy and antagonism, respectively), including SynergyFinder (Ianevski *et al.*, 2017). For analysis of very large datasets that are typically generated by high-throughput drug combination screens, we prefer to use bayesynergy (Rønneberg *et al.*, 2021), which uses a Bayesian semi-parametric model that analyzes the volume under the surface to calculate synergy scores. Bayesynergy is flexible, in that it allows the estimation of several relevant measures of interest including synergy and antagonism, but also for example total efficacy. As a fully probabilistic model bayesynergy handles uncertainty in these estimates correctly, which allows proper control of the expected proportion of false positive results, a key requirement for large screens. The synergy scores can be used to generate several plots that visualize synergistic and antagonistic relationships between drugs (Fig. 1C and Supplementary FigS S18 and S19).

### 2.5 Documentation and extended development

Screenwerk is implemented using R. Both user documentation and technical documentation are available for the use and implementation of the R-package. The R-package is available as an open-source project under GPL (GNU General Public License) v3.0 and open for further developmental contribution at https://github.com/Enserink-lab/screenwerk; the R source code is publicly available at https://github.com/Enserink-lab/screenwerk under GNU General Public License v3.0; and bayesynergy is accessible at https://github.com/ocbe-uio/bayesynergy. Selected modules are available through Galaxy, an open-source platform for FAIR data analysis at https://oncotools.elixir.no.

More details, including an example of screenwerk analysis, are available in the Supplementary Information.

## 3 Conclusion

Screenwerk provides an end-to-end pipeline for drug combination screening, ranging from design of the drug combination library to generation of drug interaction files. It integrates quality control procedures and quantifies relative drug interactions. The modularity of screenwerk allows for coupling of separate modules to various forms of input data and drug interaction applications. The possibility of visualizing data at multiple steps of the drug combination screen assists with identification and correction of potential technical issues and provides an intuitive overview of the drug interaction landscape.

## Supplementary Material

btac840_Supplementary_Data
