# Supplementary material for: screenwerk: a modular tool for the design and analysis of drug combination screens
Source: Bioinformatics. 2022 Dec 27;39(1):btac840. doi: 10.1093/bioinformatics/btac840 (PMC9825784; doi:10.1093/bioinformatics/btac840)
Supplement: btac840_Supplementary_Data [file btac840_supplementary_data.zip › screenwerk_supplementary.docx]

**Supplemental information**

Detailed description of screenwerk.

## Pre-experimental: setting-up the experiment and generating dispensing data

## Generating a list of drug concentrations (*doses*)

To be able to designate combinations of drugs in a drug combination screen, an inventory list of drug names and concentrations needs to be provided. This list can be provided in a wide data format, which is readable for humans and intuitively used by many users, and provided in form of a comma-separated values (CSV) file. Each dose for a given drug is provided in the form of the example shown in Suppl. Table S1.

**Suppl. Table S1. Example of a list of drugs and doses.**

| **Number** | **Drug** | **6^th^ Dose** | **5^th^ Dose** | **4^th^ Dose** | **3^rd^ Dose** | **2^nd^ Dose** | **1^st^ Dose** | **Unit** |
| --- | --- | --- | --- | --- | --- | --- | --- | --- |
| 2 | Venetoclax | 60 | 30 | 7,5 | 1,875 | 0,46875 | 0,1171875 | μM |
| 13 | Methotrexate | 0,3 | 0,15 | 0,0375 | 0,009375 | 0,00234375 | 0,0005859375 | μM |
| 23 | Cladribine | 10 | 5 | 1 | 0,2 | 0,04 | 0,008 | μM |
| 24 | Everolimus | 20 | 10 | 1 | 0,1 | 0,01 | 0,001 | μM |
| 28 | Cytarabine | 10 | 5 | 1 | 0,2 | 0,04 | 0,008 | μM |

There are no restrictions to the number of doses that can be used. The labels for each dose are free to choose, but must contain the word “dose” or have the identifier for the doses declared through the respective argument. Each drug is provided as a single line.

The function can be called

**generateListofDoses**(listofDoses, .doseIdentifier = "dose", .dropCol = TRUE)

The function checks for conformity of the list and allows any non-essential columns to be dropped, with the argument .dropCol.

The output from the function above is a list of drug concentrations in a long data-format, which can then be used to combine drugs at individual drug concentrations with each other.

### Generating a list of combinations

This module will generate a list of combinations from the list of drugs and drug concentrations provided. This list will then be used to generate the dispensing layout and files.

**combineDrugs**(listofDoses = file.path(inst/extdata/library/listofdoses.csv), .combineDoses=c(2:5), .noReplicates = 3, .drugRepAttrib = "single")

The generated list of combinations will look as shown in Suppl. Table S2.

**Suppl. Table S2. Example of a list of drug combinations**

| **Drug.1** | **Dose.1** | **Unit.1** | **Drug.2** | **Dose.2** | **Unit.2** |
| --- | --- | --- | --- | --- | --- |
| Venetoclax | 30 | μM | Cytarabine | 5 | μM |
| Methotrexate | 0.15 | μM | Venetoclax | 30 | μM |
| Cytarabine | 10 | μM | Cytarabine | 10 | μM |
| AG-221 (Enasidenib) | 50 | μM | Ponatinib | 2 | μM |
| Methotrexate | 0.15 | μM | AG-221 (Enasidenib) | 12.5 | μM |

This function uses the *list of doses* generated in the previous step and allows for selection of individual drug doses to be combined. In this example, a dose range of in total 6 concentrations for each drug is being used, but only the four innermost doses are combined, i.e. the second lowest up to the fifth dose. This is achieved by providing the dose range through the argument .combineDoses = c(2:5).

In addition, technical triplicates can be chosen through the argument .noReplicates = 3, specifically for single drug treatments, drug combinations, or both, using the argument .drugRepAttrib = “single”.

After a list of combinations is obtained, additional files need to be provided, which include a *list of drugs*, *volumes*, *controls* and *stock concentrations*, respectively. The *list of drugs* contains a unique id or number, the drug name and its CAS number (Suppl. Table S3).

**Suppl. Table S3. Example of a reference list of drugs.**

| **NUMBER** | **NAME** | **CAS_NUMBER** |
| --- | --- | --- |
| 2 | Venetoclax | 1257044-40-8 |
| 13 | Methotrexate | 1959-05-02 |
| 23 | Cladribine | 4291-63-8 |
| 24 | Everolimus | 159351-69-6 |
| 28 | Cytarabine | 147-94-4 |

The *list of volumes* contains the volumes at which each dose is being dispensed from the source plate to the destination plate (Suppl. Table S4). This list resembles and follows the same formatting of the *list of doses* that was provided earlier (Suppl. Table S1). Due to the limitations of the acoustic liquid dispenser all volumes were dispensed at increments of 2.5 nl.

**Suppl. Table S4. Example of a list of volumes.**

| **Number** | **Drug** | **Vol 6th Dose** | **Vol 5th Dose** | **Vol 4th Dose** | **Vol 3rd Dose** | **Vol 2nd Dose** | **Vol 1st Dose** | **Unit** |
| --- | --- | --- | --- | --- | --- | --- | --- | --- |
| 2 | Venetoclax | 10 | 5 | 5 | 5 | 5 | 5 | nl |
| 13 | Methotrexate | 10 | 5 | 5 | 5 | 5 | 5 | nl |
| 23 | Cladribine | 10 | 5 | 5 | 5 | 5 | 5 | nl |
| 24 | Everolimus | 10 | 5 | 5 | 5 | 5 | 5 | nl |
| 28 | Cytarabine | 10 | 5 | 5 | 5 | 5 | 5 | nl |

Finally, the *list of stock concentrations* lists the stock concentrations of the drugs used (Suppl. Table S5).

**Suppl. Table S5. Example of a list of stock concentrations.**

| **NUMBER** | **DRUG** | **CONCENTRATION** | **UNIT** |
| --- | --- | --- | --- |
| 2 | Venetoclax | 100 | mM |
| 13 | Methotrexate | 200 | mM |
| 23 | Cladribine | 100 | mM |
| 24 | Everolimus | 30 | mM |
| 28 | Cytarabine | 200 | mM |

### The exclusion of individual wells from an experimental set-up

A complementary module of this package allows for generation of a list of individual wells or series of wells that should be excluded from the experimental set-up, for instance to exclude certain wells on multi-well microplates that are sensitive to evaporation. This function supports the most common microplate formats, ranging from 6 to 1536 wells. It allows for selection of a combination of designated rows, columns and individual wells for exclusion. Rows and columns can be specified by their names, which are designated letters for rows (A, B, C, ..) and designated numbers for columns (1, 2, 3, ..). Alternatively, in addition to entire rows and columns, individual wells (A1, B2, C3, ..) can be marked for exclusion. The nomenclature of wells follows the ANSI guidelines set by the Society for Laboratory Automation and Screening (Society for Laboratory Automation and Screening). In addition, the function offers the convenient possibility to exclude the outer wells of any given plate format.

**excludeWells**(1536, outer.wells = TRUE)

The function will generate a list of well coordinates that can be used to specifically exclude wells from the dispensing procedure. The function can also be used in a more general manner to generate any sequence of wells for various analytical or experimental tasks, such as generating a list of wells of interest that can be used to specifically select or filter for individual wells, rather than for exclusion.

### The import of plate maps

This step offers a module that allows the user to import plate maps from various sources. These plate maps can be directly imported from a comma separated values (.csv) file, or by utilizing a third-party software, such as the IncuCyte® Plate Map Editor. Third-party applications and tools usually work with proprietary file formats, which are not directly usable with R.

The function importPlateMap allows the user to import IncuCyte® PlateMap files, which are in a proprietary file format used by the IncuCyte® Plate Map Editor. This allows users for designing source plates using the IncuCyte® Plate Map Editor and importing the plate map into the pipeline. This module can also be used outside the scope of drug sensitivity screens, e.g. for projects that utilize the IncuCyte® live cell imaging system.

**importPlateMap**(importFile = file.path(inst/extdata/library/), .fileFormat = ".PlateMap", .sourcePlateConv = TRUE)

The plate maps can be imported either from a single file or from multiple files. It is recommended to specify the file format of the file to import with the argument .fileFormat, especially if the folder from which the files are imported contain files with different file formats. Alternatively, the function is able to detect any of the supported file formats automatically and import them. With the argument .plateMapGrp individual groups of cells, drugs or conditions can be defined. The argument .sourcePlateConv allows for converting the file format of the plate map to only the essential columns required for the generation of the dispensing files by removing all non-essential data, such as ambiguous columns or empty rows.

A schematic representation of a source plate is shown in Suppl. Figure S1 with each dose used in the drug sensitivity screen located in an individual well. Additional controls can be included on a separate source plate, containing the positive (BzCl) and negative (DMSO) control in multiple wells with enough volume for a given number of experimental set of plates.


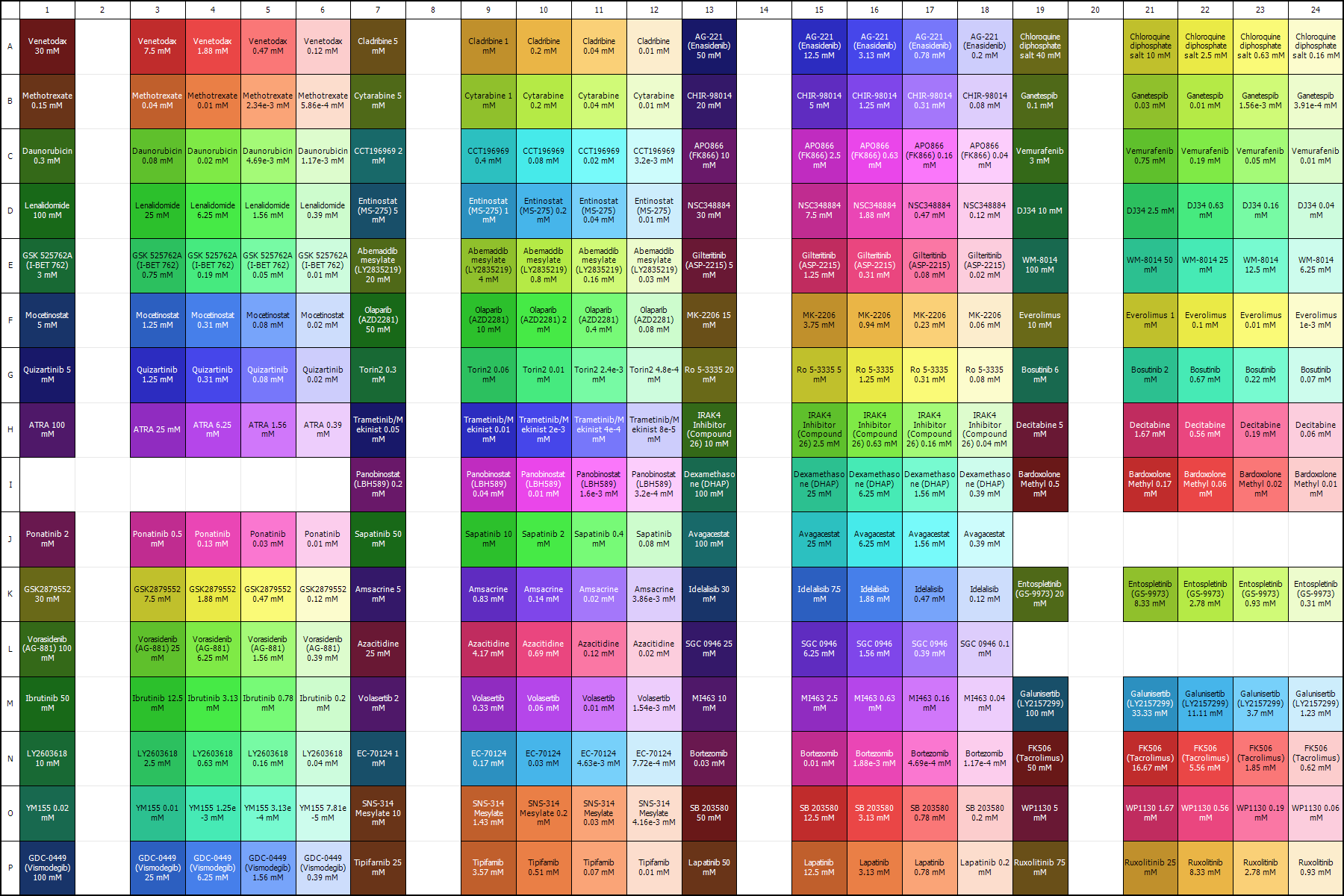


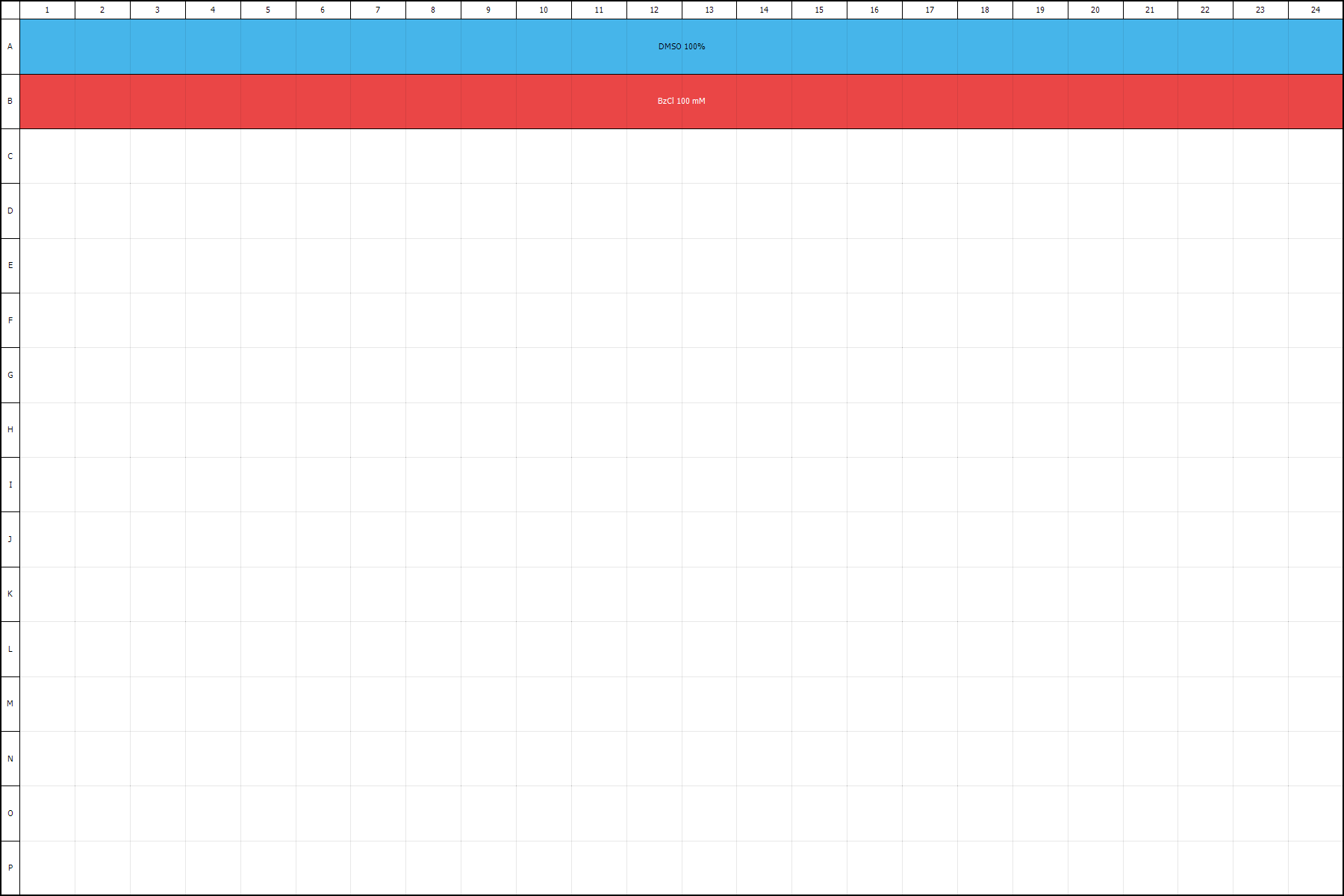


**Supplementary Figure S1.** Examples of a source plate with the stock concentrations of drugs (*top*) and a control plate (*bottom*).

### generating dispensing files

It is crucial to the overall design and execution of the assay to generate a dispensing file that contains instructions regarding which drugs should be dispensed at which concentration from source plates holding drug stocks onto destination plates that will be used for the actual assay.

With this module it is possible to generate a plate layout in which the individual single drug treatments and drug combinations are distributed across a number of plates. It takes into account the controls and excluded wells that have been previously defined. It is also possible to generate dispensing files in a predefined format specific for (various) dispensing robots. At the moment the LABCYTE Echo 550 acoustic dispenser is supported (more dispensers can be integrated at a later time point). Due to the technical properties of the Echo acoustic dispenser, usually only volumes of 2.5 nl increments can be dispensed, which needs to be taken into account when designing the source plate with the appropriate stock concentrations of drugs. This technical limitation can be accommodated by providing a list of volumes to be dispensed from the source plate to obtain the desired final drug concentration of a given treatment, when generating the dispensing files. The list of the volumes serves consequently as a reference between the stock concentration on the source plate and the final concentrations on the destination plate. This allows for the transfer of a drug from a single well on the source plate to the destination plate at any given volume, instructing the robot which well on the source plate and which well on the destination plate should be used. Future work will allow a more customizable approach, in which individual dilutions can be dispensed from individual or multiple wells.

Furthermore, a list of combinations, a list of controls, and a list of excluded wells is required to generate a dispensing file. Depending on the experimental set-up, it is possible to exclude certain wells from being targeted to avoid technical and experimental variation, such as the edge effect on most microtiter plates due to a higher evaporation on the outer wells. In addition, a list of wells or a plate map of the source plate is required. The source plate contains the drugs in their stock concentrations to be dispensed in dilutions onto the destination plate. This module is able to read the proprietary plate map file format from the Incucyte® ZOOM Live-Cell Analysis System from Essen BioScience. Alternatively, a reference list of wells on the source plate can be provided separately.

The final dispensing layout on the destination plate can arrange sample and drug treatments in sequential order, or as recommended, randomized across a set of plates. In addition, the number of sets can be specified in case several drug sensitivity screens will be carried out with multiple patient samples or cell lines. For each dispensing and drug combination a unique identifier is given, which is linked to a unique coordinate on the destination plate.

Additional features of this module include the visualization of the final plate layout. In cases where the dispensing volume needs to be monitored in order to avoid critical levels and the depletion of drugs, the module is able to provide feedback on the dispensed volume per set as well as the total volume for a given source plate.

**generateDispensingData**(listofCombinations, listofDrugs, listofDoses,

listofVolumes, listofCtrls, listofStockConcentrations,

sourcePlate, listofExWells, .ctrlReplicates = 8,

.addUntreated = list(name = "Untreated", replicates = 8),

.finalWellVolume = 5, .plateFormat = 1536,

.destinationPlateID = "0920",

.randomizeDispensing = TRUE, .probeDispensing = FALSE)

The requirements for this function are relatively extensive, with a number of lists, as mentioned above. It also requires a map of the source plates and optionally a list of excluded wells. In the example above, eight technical replicates for each control, as well as an additional untreated control were designated to the experiment. Each control is then dispensed at the given number of replicates on each individual plate. Additionally, the final volume per well and the plate format with the number of wells needs to be specified. Each experiment has a unique id, which is used to label each set of destination plates. There is also a possibility to randomize all drug treatments across a set of plates.

The function will generate an R-object of class S3:dispensingData, with all the input data retained.

A helpful feature is the possibility of a dry-run without actually generating any dispensing data. The dry-run provides a short summary and allows for estimation of the number of drugs, controls, and plates among other things that were required for a given drug screen.

The same can also be achieved once the dispensing data was generated through the use of the *summary* function. This can especially be beneficial if an experimental design needs to be repeated after a period of time without remembering the exact details of the design, or if an experimental design was provided by another lab.

**summary**(dispensingData)

The function produced the following summary:

Summary for dispensing ID: 0920

Number of drug treatments: 30 378

Number of unique drug combinations: 29 280

Number of unique single drug treatments: 366

Number of excluded wells per plate: 156

Number of controls per plate: 24

Number of total plates: 23

Number of drugs: 61, Number of doses: 6/4

We can see from above that in this example the experiment required a total number of 23 1536-well plates for a single experimental sample with 30 378 drug treatments. In this case a total of 6 doses per drug are being used of which 4 doses are being combined.

In cases where only the dispensing data needs to be used, such as for any other data processing, the function *print* will extract only the dispensing data, without any metadata other any of the initial data sets.

**print**(dispensingData)

The function prints the dispensing file as shown in Suppl. Table S6.

**Suppl. Table S6. An example of a dispensing data set.** The headers are labeled as followed: [1] Combination.ID, [2] Sample Name, [3] CAS number, [4] Drug Concentration, [5] Unit, [6] Transfer Volume, [7] Source Plate Barcode, [8] Source Well, [9] Destination Well, [10] Destination Plate Barcode, [11] Plate Number

| **[1]** | **[2]** | **[3]** | **[4]** | **[5]** | **[6]** | **[7]** | **[8]** | **[9]** | **[10]** | **[11]** |
| --- | --- | --- | --- | --- | --- | --- | --- | --- | --- | --- |
| 1 | NSC348884 | 81624-55-7 | 0,46875 | μM | 5 | C010 | D17 | E5 | 0920 | Plate22 |
| 2 | Avagacestat | 1146699-66-2 | 25 | μM | 5 | C010 | J15 | H25 | 0920 | Plate11 |
| 3 | GSK2879552 | 1401966-69-5 | 7,5 | μM | 5 | C010 | K3 | S36 | 0920 | Plate15 |
| 4 | CHIR-98014 | 252935-94-7 | 5 | μM | 5 | C010 | B15 | U41 | 0920 | Plate22 |
| 5 | CHIR-98014 | 252935-94-7 | 20 | μM | 5 | C010 | B13 | I16 | 0920 | Plate21 |

In addition, the dispensing data can be visualized on the basis of a plate map using the function *plot* (Suppl. Fig. S2).

**plot**(dispensingData, .saveto)

This will plot each plate of a given set and save it as an image/png file, if requested.


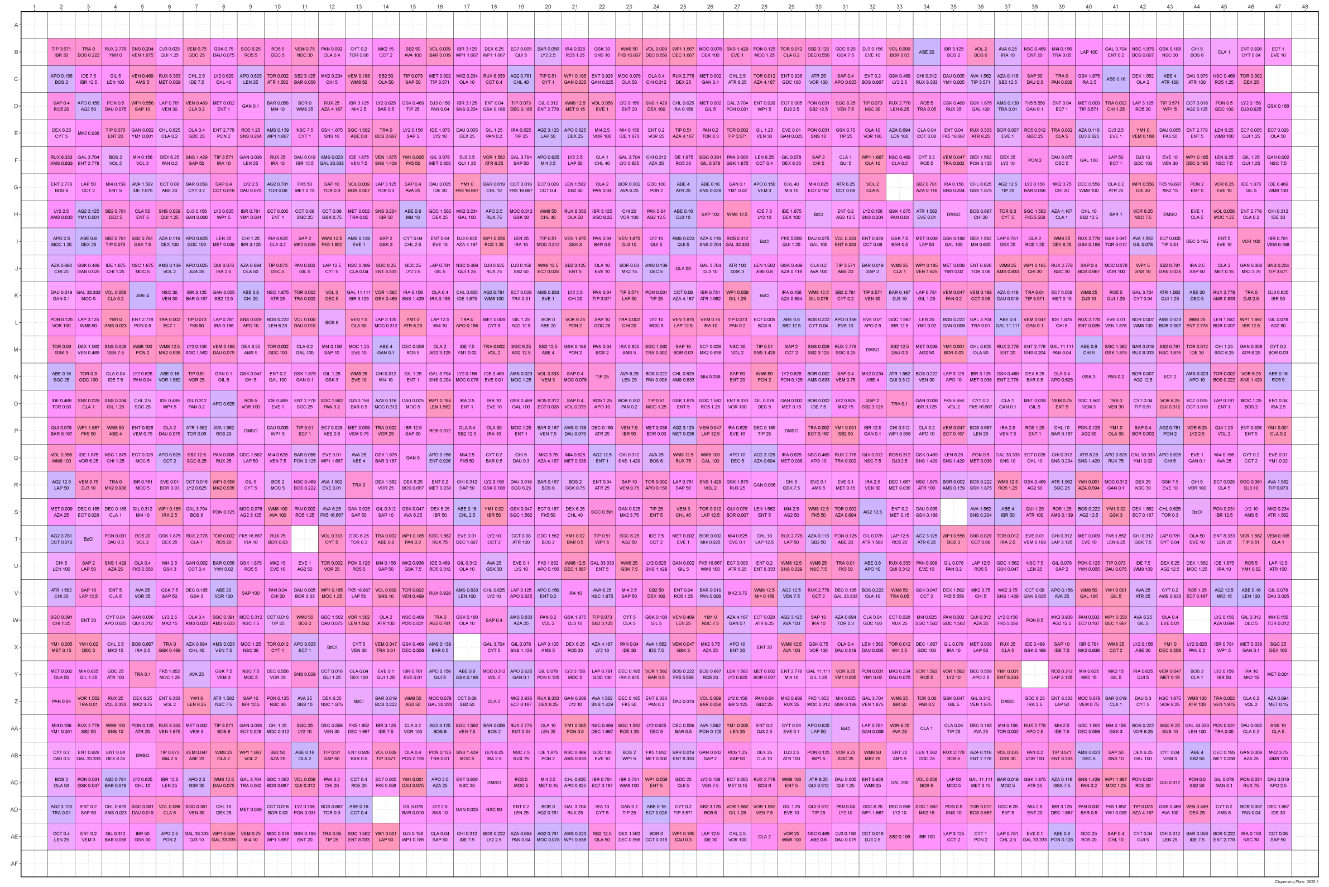


**Supplementary Figure S2.** Plate map showing one of 23 destination plates with randomized drug combination treatments, single drug treatments and eight positive, negative and untreated controls.

Finally, the dispensing data can be saved to a set of files, which were used to instruct the dispensing robot. This can be achieved using the function *save*.

**save**(dispensingData, .saveto, .sets = 26, .labels = "alphabetic", .split = FALSE, .format = "Echo")

In the example above a set of 26 plates are being generated, one for each experiment with a different cell line. Each set can be labelled in alphabetic sequential order (A, B, C, … AA, AB, AC) and generated into a single dispensing file. Alternatively, it is possible to split the dispensing data to individual files for each set. In the same example an Echo Acoustic Liquid Handler (E5XX-1366, Echo 550) from Labcyte Inc., Beckman Coulter Life Sciences, is being used and the data format has to be selected accordingly.

**Suppl. Table S7.** An example of a dispensing file.

| **Sample Name** | **Source Plate Barcode** | **Source Well** | **Transfer Volume** | **Destination Well** |
| --- | --- | --- | --- | --- |
| NSC348884 | C010 | D17 | 5 | E5 |
| Avagacestat | C010 | J15 | 5 | H25 |
| GSK2879552 | C010 | K3 | 5 | S36 |
| CHIR-98014 | C010 | B15 | 5 | U41 |
| CHIR-98014 | C010 | B13 | 5 | I16 |

## post-experimental: reading raw data and consolidating datasets

screenwerk offers modules to read the raw data and consolidate datasets prior to any analysis. These modules are utilized post experimentally and include various modules for data handling, analysis and data reporting.

### reading raw data files

With this module the raw measurements are read from multiple files and formatted for data processing, which is necessary for downstream analysis. At the moment different output formats and files are supported, among them files from the *PerkinElmer EnVision* and *Victor X Multimode Microplate Reader*, as well as the possibility to read the raw measurements directly with a layout representing the well plate format independent of a specific machine or manufacturer. Due to its modular build, this module can be extended with file formats from additional plate reading devices at any given time.

To be able to correctly associate each plate with the exported raw data, the name of each file had to contain the specific plate barcode, or a unique plate identifier.

**readRAWData**(.readfrom = file.path(inst/extdata/raw/), .fileformat = c(".csv", ".txt"), .format = "EnVision")

Even though the raw measurement files were exported as csv files, both file formats can be stated in the function as a premeditative measure. If the experimental plates were read with an EnVision multimode plate reader, the format can be specified accordingly. This function is capable of detecting the export-format and identifying the used text delimiter, such as comma-, semicolon- or tab-separated. It also automatically detects the plate format and identifies the raw data among all the metadata.

The output only contains the raw measurements in reference to the plate and well number, without being associated with any treatments or samples (that step is performed by a separate module). This function only reads data from microplate readers and can be used for a number of different studies.

### consolidate Data

Before the raw measurements can be used for downstream analysis of drug sensitivities, a final reference data set needs to be built. This can be achieved by consolidating the raw measurements with the generated dispensing data that was used to run the experiments. This module associates the raw measurements with the dispensing data. Its main purpose is to build a complete data set with all the raw measurements in reference to the individual drug treatments on all the plates, which is assigned a unique combination identifier and contains the complete set of meta data, such as plate number and plate barcode, drug identifiers (CAS no.), sample type, source and destination wells, transfer volumes etc. The output from this module ensembles the first consolidated full data set formatted for downstream processing and analysis. The integrity of the data set is maintained and is not subject to changes during any of the downstream processes, but is rather used as a master dataset for different derivatives.

Before this can be done, it is necessary to import a barcode reference list with the names of the samples used in the drug screen and by associating them to the corresponding plate id and set.

barcodeReference <- read.csv(file=file.path(“inst/extdata/library/platebarcode.csv"),

check.names=FALSE, header=TRUE, stringsAsFactors=FALSE,

colClasses=c("PlateID"="character"), comment.char = "#",

blank.lines.skip = TRUE, na.strings = "", sep = ",",

dec = ".", nrows = 1, skip=0)

The barcode reference can be imported from a csv file prior to consolidation of both data sets.

**Suppl. Table S8.** An example of a plate barcode reference list

| **PlateID** | **Set** | **Number** | **Sample** |
| --- | --- | --- | --- |
| 0920 | A | 23 | MeWo |

Once this information is provided, the raw measurements can be consolidated with the dispensing data, using the function below:

**consolidateData**(dispensingData, rawMeasurements, barcodeReference)

Since this function is dependent on datasets previously generated, the data needs to be of a specific class to ensure data integrity. The **dispensingData needs to** an object of class *'dispensingData'*, while the **rawMeasurements** an object of class *'rawMeasurements'*. The .barcodeReference is a list of samples for each set of plates.

The consolidated data can also be exported for use outside the scope of screenwerk with the function *save*.

**save**(consolidatedData, .saveto, .fileformat = “.csv”, .format = FALSE)

The exported data can then be processed further and used for downstream analysis with any other tool. In addition, this function allows to export the data specifically for the use with Breeze (Potdar *et al.*, 2020) by setting the argument . format = “breeze”.

Once both the dispensing data and the raw measurements are consolidated, it is possible to perform the first analysis by running the quality control (QC).

### QC (quality control)

The package comes with a set of quality control (qc) tools that are imperative for the quality assurance of a drug sensitivity screen. This module provides a function that offers a set of quality assessments by looking at the variance of individual controls, reporting the Z’-factor (Zhang *et al.*, 1999) between the positive and negative controls, as well as looking at the signal of empty and untreated wells.

**qc**(consolidatedData, .ctrls = c("BzCl", "DMSO", "Untreated"), .qcMethod = “all”)

One of the quality methods of the function *qc* is used to assess the quality of a drug screen by looking at the variance and signal distribution between individual controls. The argument .qcMethod is used to select between individual quality assessments, or alternatively run multiple or all quality methods at once. At the moment the following qc methods are available:

*variance* : assessing the variance between individual controls both, across all plates, as well as by individual plate *emptywells* : assessing the signal of empty and untreated wells, this will also include any excluded wells *firstcolumn* : assessing the signal of wells in the first column of each plate *zprime* : assessing the distribution between the positive and negative controls.

The function returns an object of class S3:controlData, with the data and plots for each method. The data structure is looks as this:

| ▼ qcdata | list [1] (S3: controlData) | List of length 1 |
| --- | --- | --- |
| ▼MeWo | list [5] | List of length 5 |
| ► data | list [329 x 14] (S3: data.frame) | A data.frame with 329 rows and 14 columns |
| ▼ variance | list [3] | List of length 3 |
| ► data | list [3] | List of length 3 |
| ► boxplot | list [3] | List of length 3 |
| ► boxplot-byplate | list [3] | List of length 3 |
| ► empty-wells | list [23] | List of length 23 |
| ► first-column | list [9] (S3: gg, ggplot) | List of length 9 |
| ▼ z-factor | list [2] | List of length 2 |
| ► data | list [23 x 4] (S3: data.frame) | A data.frame with 23 rows and 4 columns |
| ► plot | list [9] (S3: gg, ggplot) | List of length 9 |

The function also generates a number of plots, providing an insight into the overall variance and noise based on the used controls. One of the plots compares the raw signal between the controls across all plates and provides a first assessment of variance within an experiment. For instance, in Suppl. Figure S3 the untreated control and the negative control (DMSO) have the same signal strength and level of variance as expected, compared to the positive control (BzCl) with a signal strength close to zero.


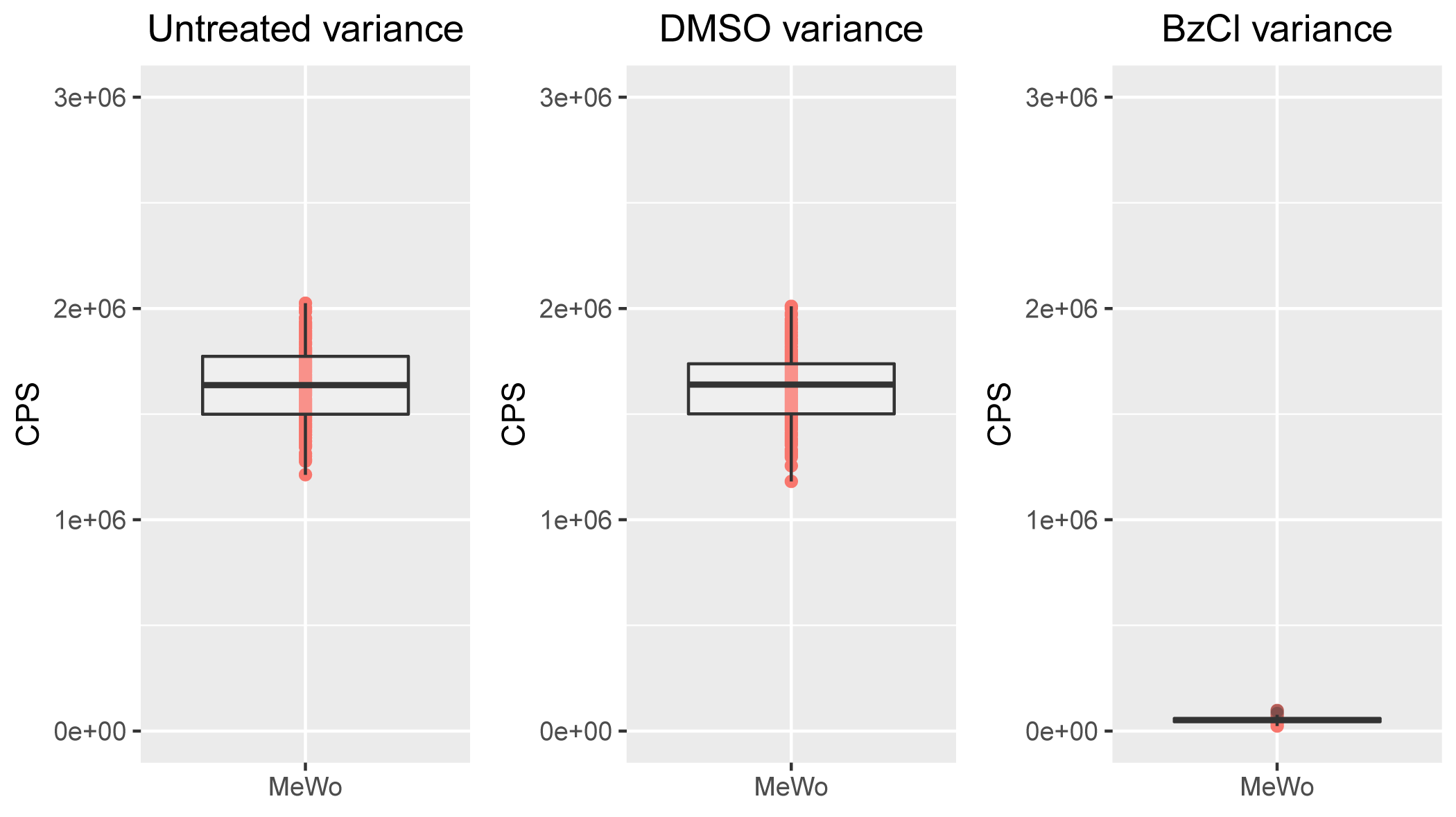


**Supplementary Figure S3.** Raw signal (CPS) distribution for each of the controls.

The variance of controls across all individual plates is also plotted to be able to identify potential irregularities on individual plates. In the example shown in Suppl. Figure S4, the pattern of signal strength and variance is similar between untreated cells and negative controls (DMSO-treated cells).


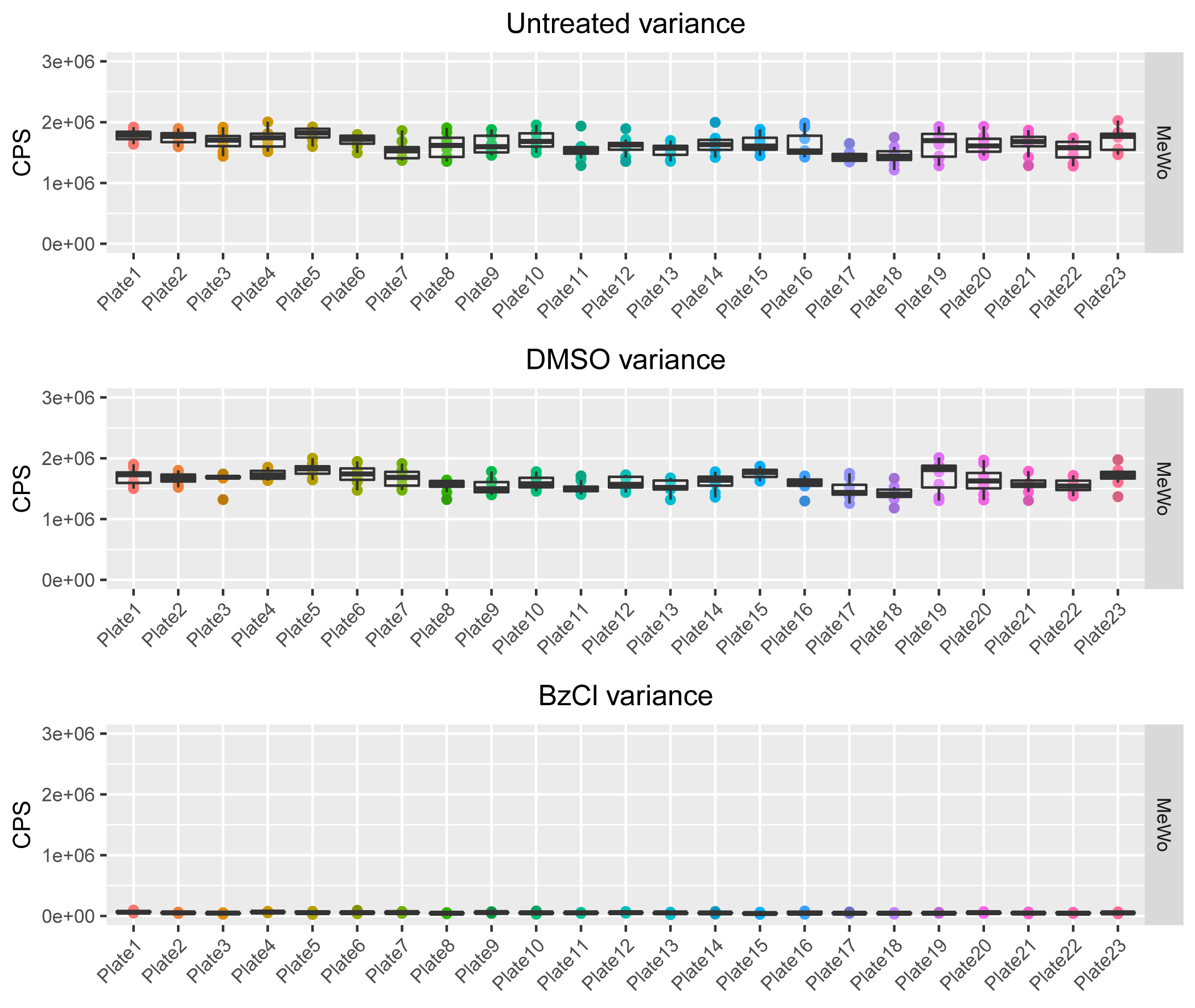


**Supplementary Figure S4.** Raw signal (counts per second, CPS) distribution for each of the controls across all plates.

Finally, screenwerk can plot variance distribution to identify potential outliers on a well to well basis (Suppl. Fig. S5). In this example, all wells outside a certain tolerance level are labeled. This allows for identification of potential technical issues in dispensing where certain wells are repetitively affected.


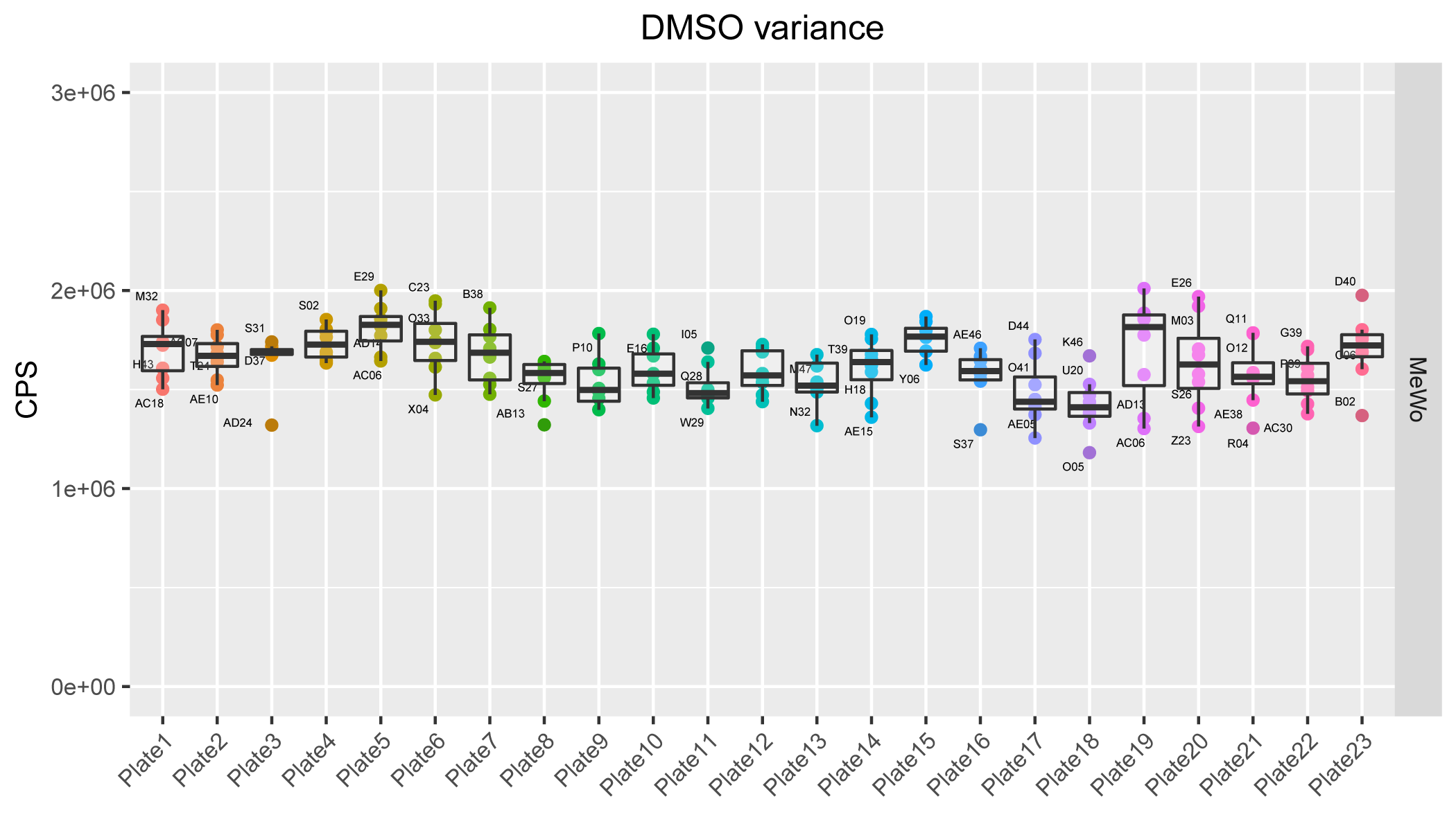


**Supplementary Figure S5.** Variance distribution of the microwell plates in which individual wells outliers with outliers have been indicated.

If desired, the signal of all the empty and excluded wells on each individual plate can also be plotted (Suppl. Fig. S6). While this is certainly not the most important function of screenwerk, it might provide insight into certain pasterns of variance across an individual plate and reveal certain dispensing issues. A variation of this plot extracts the first column of each plate and plots them side by side in order to identify potential patterns of variation across multiple plates (Suppl. Fig. S7).


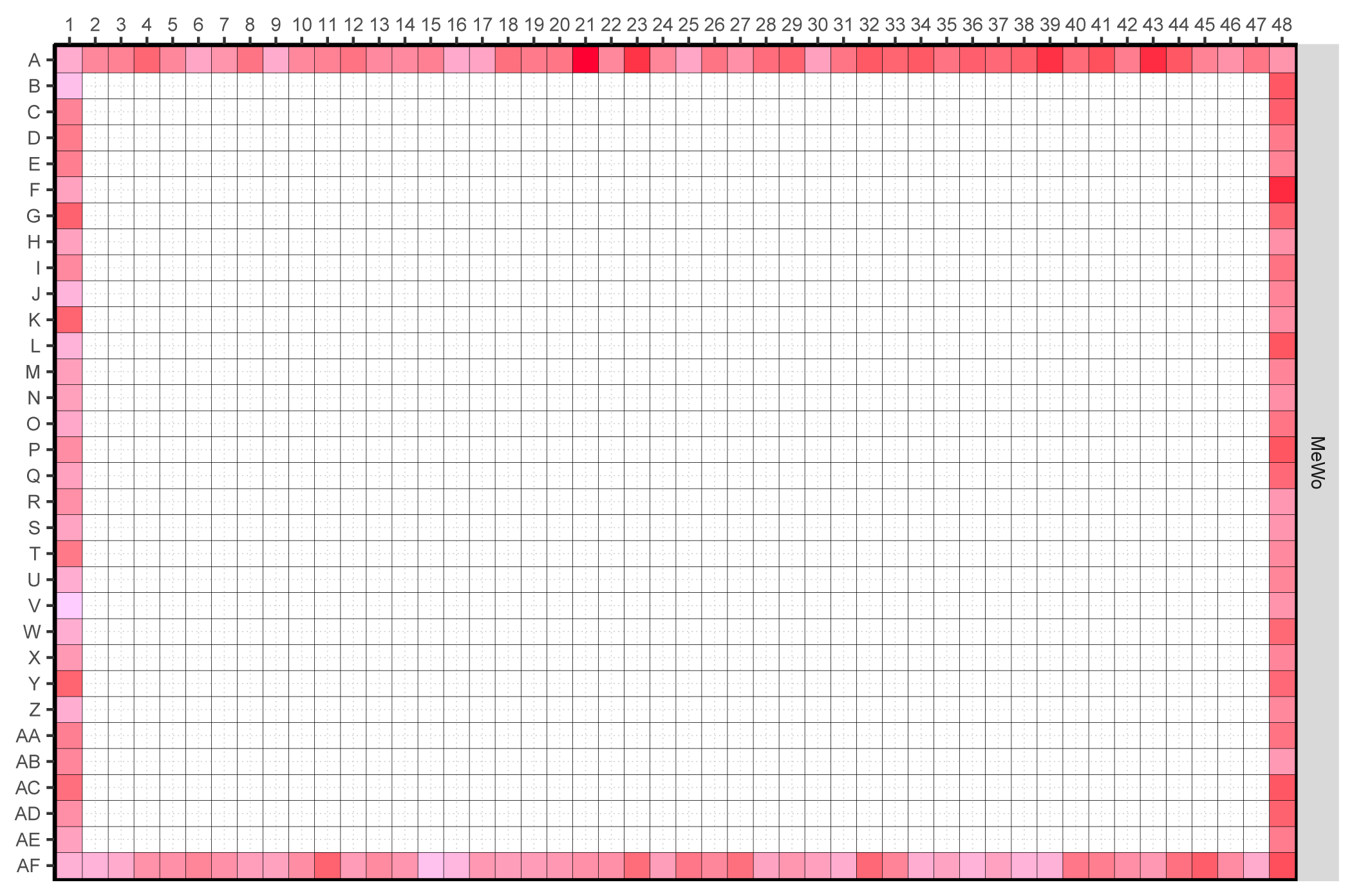


**Supplementary Figure S6.** Signal measurement of the outer (excluded) wells.


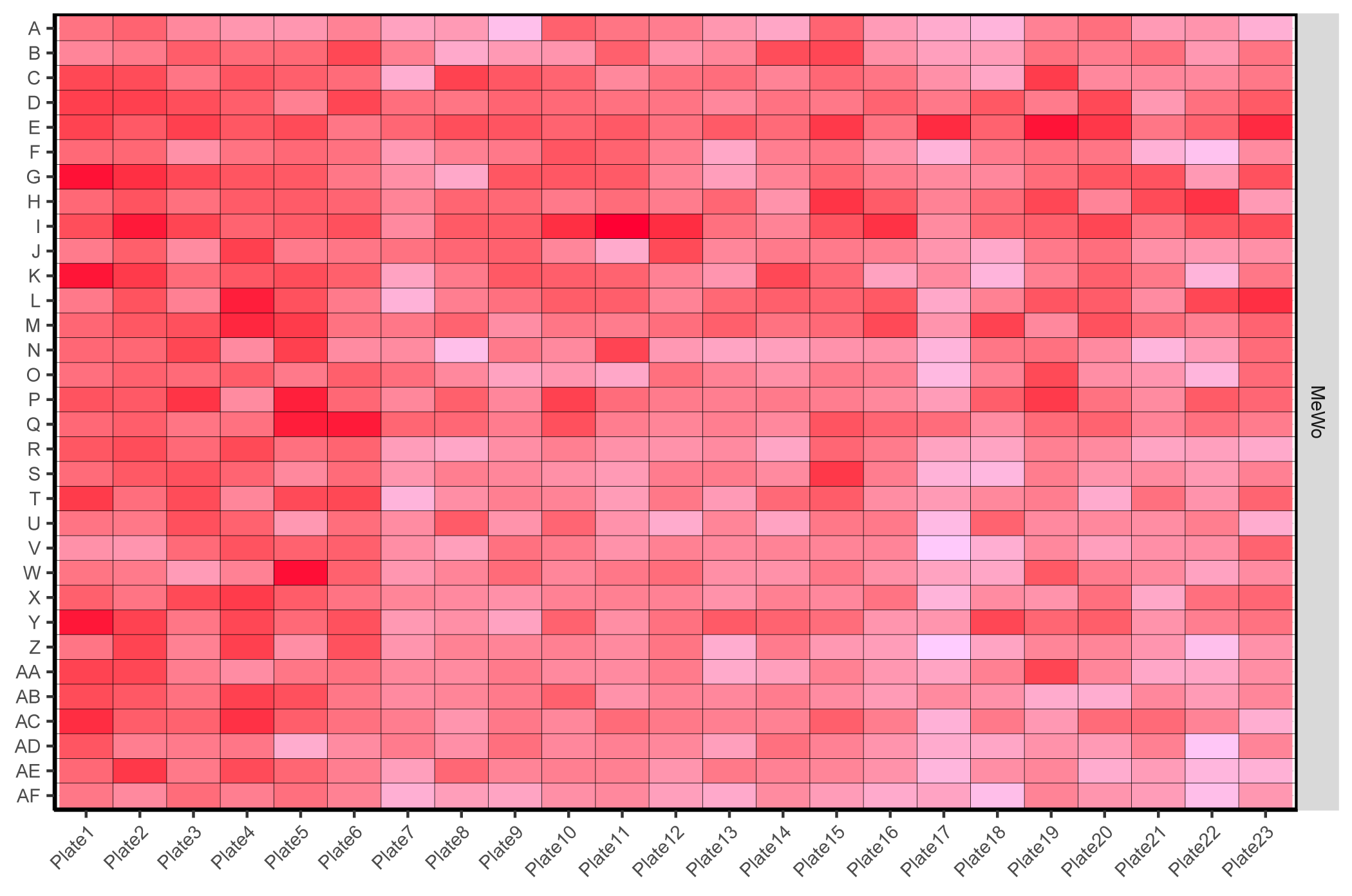


**Supplementary Figure S7.** Signal distribution of (excluded) wells in the first column of each plate.

One of the more important qc assessments is provided by *Z’-factor* (Suppl. Fig. S8). It is considered desirable that the Z’ score is above the arbitrary threshold of 0.5, which indicates a sufficiently wide separation between the positive and negative controls.


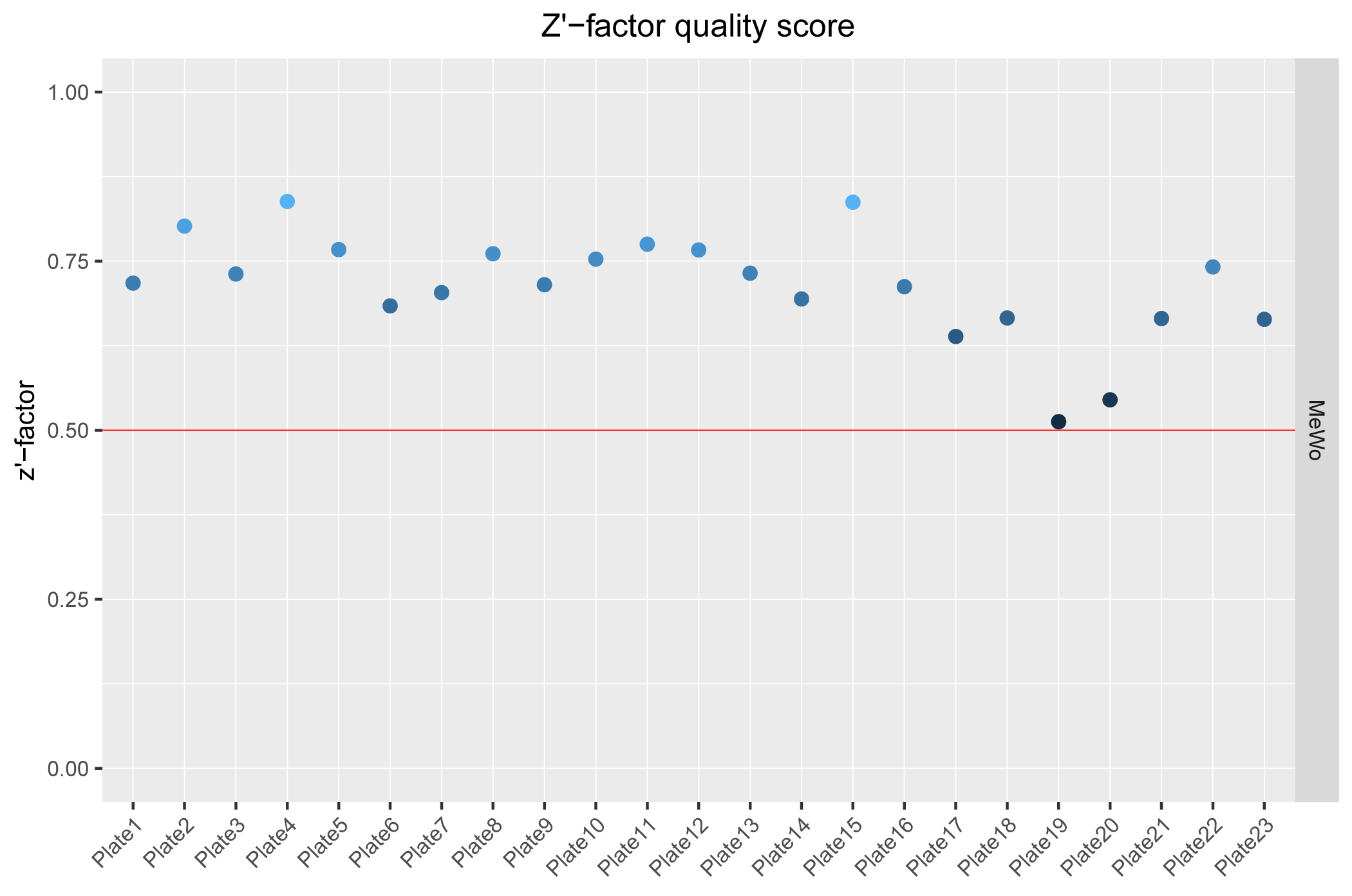


**Supplementary Figure S8.** Graph showing Z’-factors for each set of controls across all the plates.

### data processing

With this module the data is prepared for various tasks in the downstream analysis of the drug sensitivity screen. It carries out three essential steps in data processing: normalizing, splitting and assembling (re-formatting) the data.

**processData**(consolidatedData, .ctrls=list(positive="BzCl", negative="DMSO"))

The data processing unit takes advantage of the consolidated raw data and creates the first processed and modified data set for downstream analysis. This function processes the dataset by normalizing the raw measurements to the positive and negative control. It subsequently splits the data into individual datasets, i.e. for the controls, for single drug treatments, and for combination treatments. These datasets are formatted in two ways: As a list of dataframes using a tabular structure, and as a list of dose-response matrices. This is done to ensure adaptability of the dataset for different analysis tools with unique data format requirements.

### dose-response analysis

The dose-response module allows the assessment of single drug dose-responses. It gives an overall overview of the performance of individual drugs. runDRM is a function that runs the dose-response model on the single drug treatments. It performs curve fitting using a four-parameter log-logistic function (LL.4), and estimates the EC10, EC50 and EC90. The function also plots the single drug curves by viability and inhibition for each single drug.

**runDRM**(processedData, .saveto, .plot = TRUE)

The dose-response modeling is performed using the function **drm** from the R-package *drc* (Ritz *et al.*, 2015).

The function will analyze single drug treatments. It performs curve fitting using a four-parameter log-logistic function (LL.4), and estimates the EC10, EC50 and EC90. The function also plots the single drug curves by viability and inhibition for each single drug. An excerpt of the output data is shown in Suppl. Table S9, and examples of graphs in Suppl. Figure S.

**Suppl. Table S9**. A list of EC10, EC50 and EC90 estimates for each drug.

| **Sample** | **Drug** | **ED** | **Estimate** | **Std. Error** |
| --- | --- | --- | --- | --- |
| MeWo | Abemaciclib mesylate (LY2835219) | 10 | 18,5166585030146 | 139,447775779473 |
| MeWo | Abemaciclib mesylate (LY2835219) | 50 | 18,7410035107027 | 120,298549656558 |
| MeWo | Abemaciclib mesylate (LY2835219) | 90 | 18,9680666482556 | 101,30964028357 |
| MeWo | AG-221 (Enasidenib) | 10 | 11,5666411485189 | 4,96699580857896 |
| MeWo | AG-221 (Enasidenib) | 50 | 36,2896471575161 | 12,3114505023091 |


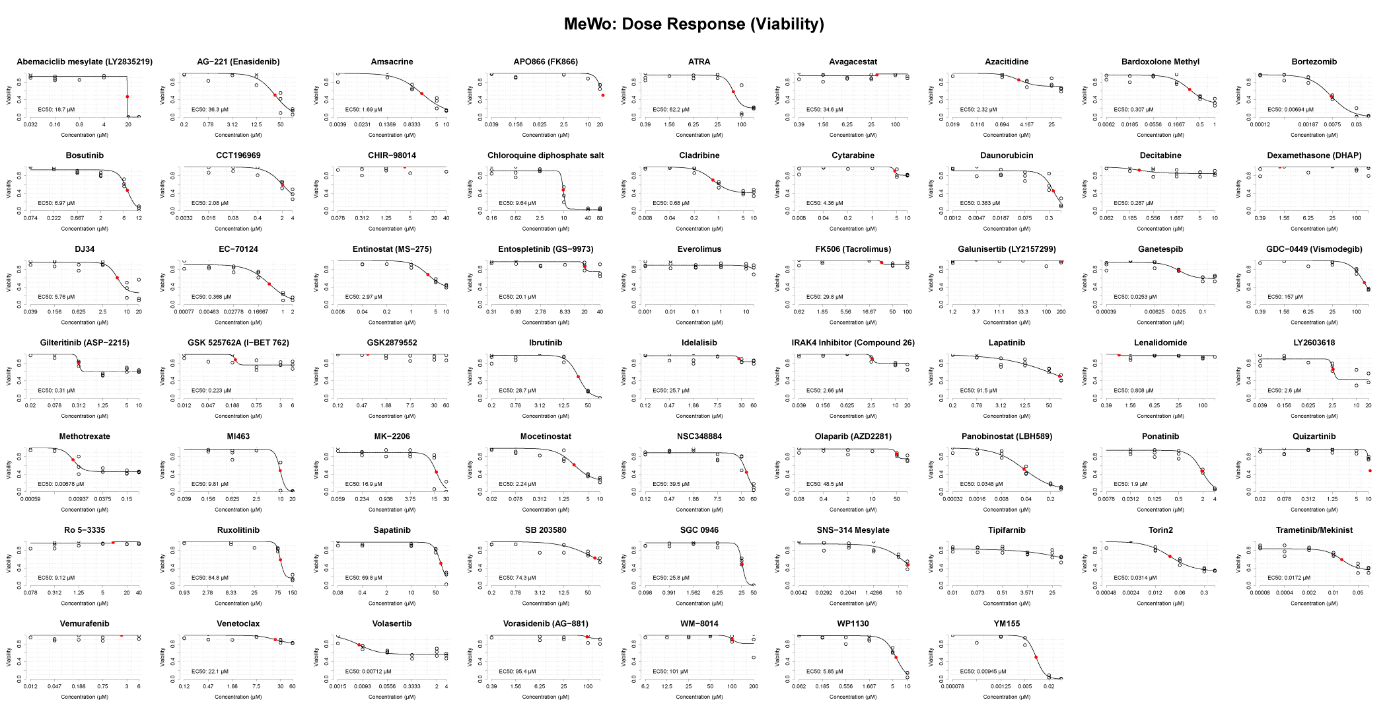


**Supplementary Figure S9.** Single drug response curves by relative cell viability for all drugs in the screen.


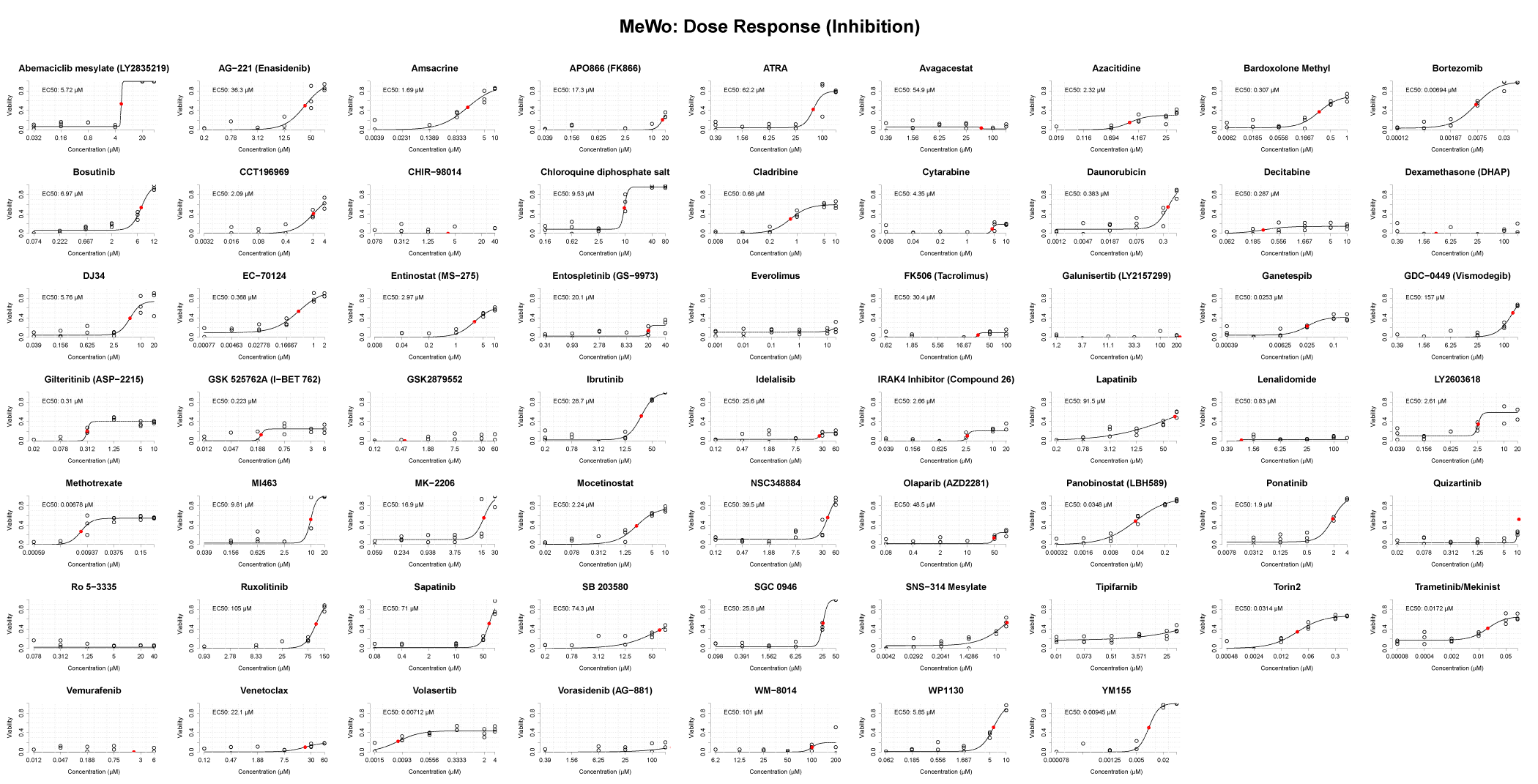


**Supplementary Figure S10**. Single drug response curves by inhibition of relative cell viability for all drugs in the screen.

In addition to the basic plots with only the dose response curves illustrated, a separate set of graphs can be generated containing the dose response matrix along with the individual dose response curves for each single drug of a given pair in a composite plot.

### custom plotting

This module generates a set of custom plots for the visualization of the drug-dose responses. customPlotting is a function that generates a set of plots that provide an overview of the dose-response not only between drugs, but also between individual samples. Furthermore, it plots the dose-response matrix for drug combinations along with the dose-response curves for each individual drug pair (see Fig. 1B for an example).

The function requires the processed data as an object of class *'processedData'*, along with the dose response data as an object of class *'doseRespModel'*.

**customPlotting**(processedData, doseRespModel, .saveto)

This function can also plot an overview of single drug responses for each sample, as well as for each drug (Suppl. Fig. S11 and 12, respectively).


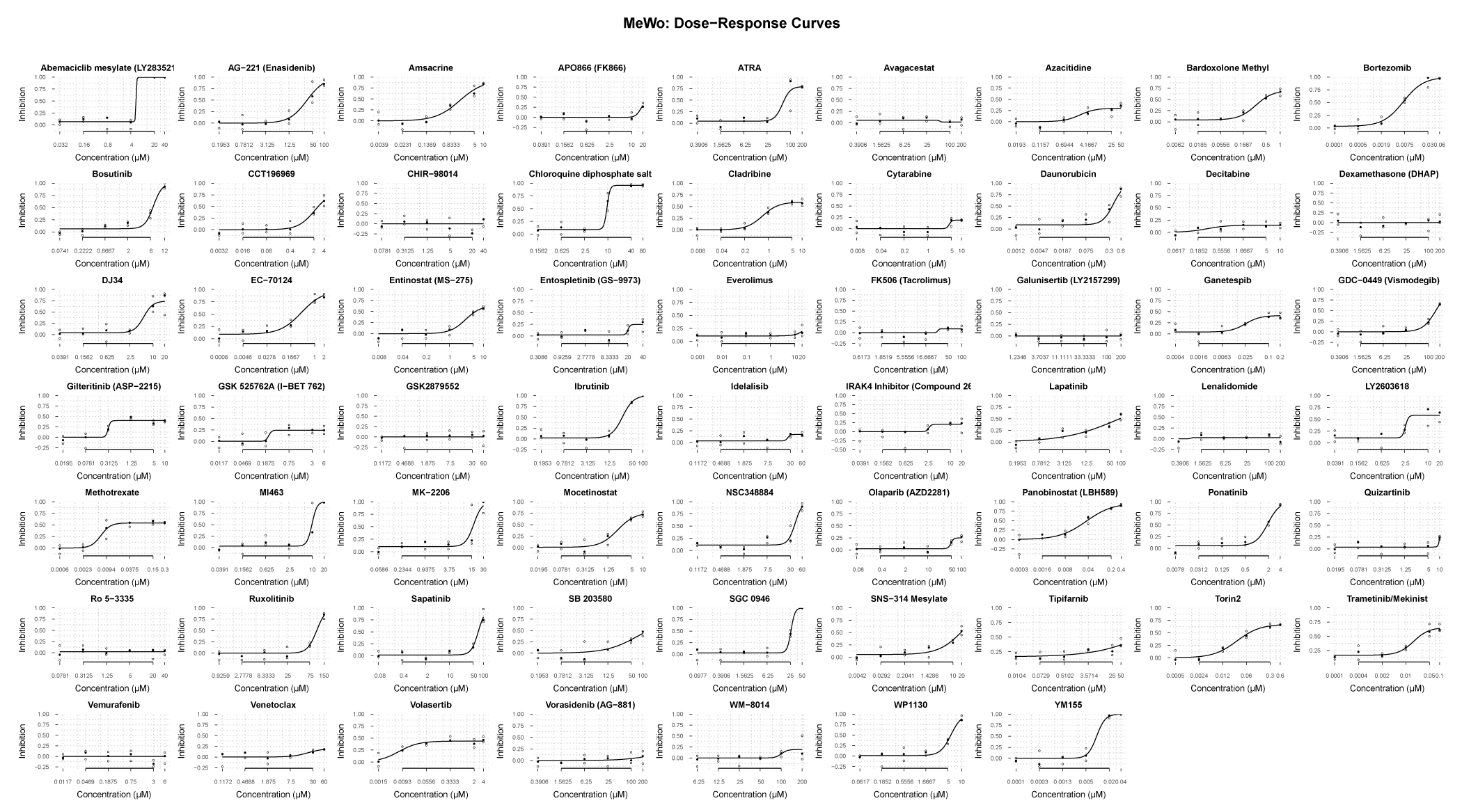


**Supplementary Figure S11.** Single drug response curves of all drugs for a given cell line.


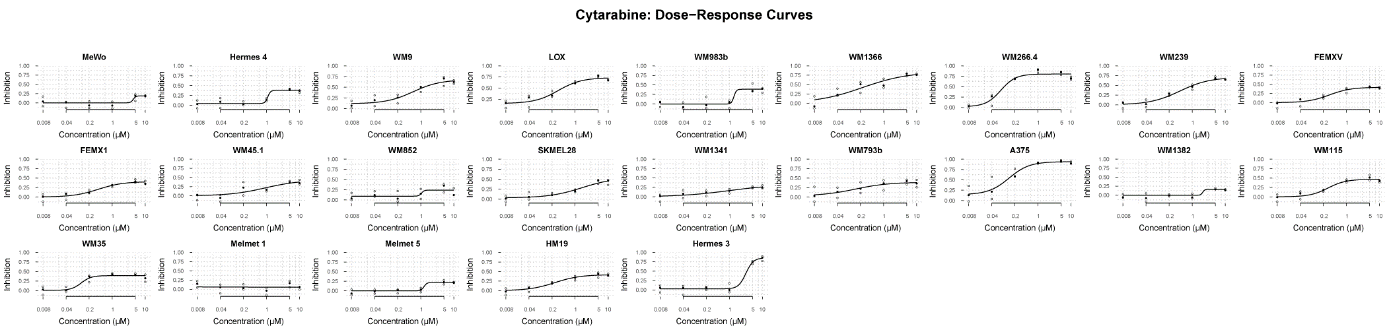


**Supplementary Figure S12.** Single drug response curves of all cell lines for a given drug.

### dynamic range

This module offers the assessment of the dynamic drug-activity range for individual drug-dose responses. The dynamic range is used to provide an assessment of the drug activity range across the chosen range of drug concentrations. This is an important indicator, particularly for drug combination screens, because it determines whether the selected drug concentration ranges used for the combination treatments do indeed fall within the activity ranges of the drugs. Here, the dynamic range is considered the dose-response range between the ED10 and ED90. This function will indicate the expected and the observed drug activity range for each drug and sample. It will generate plots based on the fitted dose-response models as well as unfitted curves. With the single drug response data, it is possible to assess the dynamic drug-activity range for individual drugs. The function estimates the dynamic drug-activity range (DDAR) across a number of doses for each drug response and generates a set of plots. The DDAR is predefined as the range between the ED10 and ED90.

**dynamicRange**(doseRespModel)

One set of plots looks at the dynamic range for each individual drug and highlights potential drugs that do not show any effect across all doses (Suppl. Fig. S13).


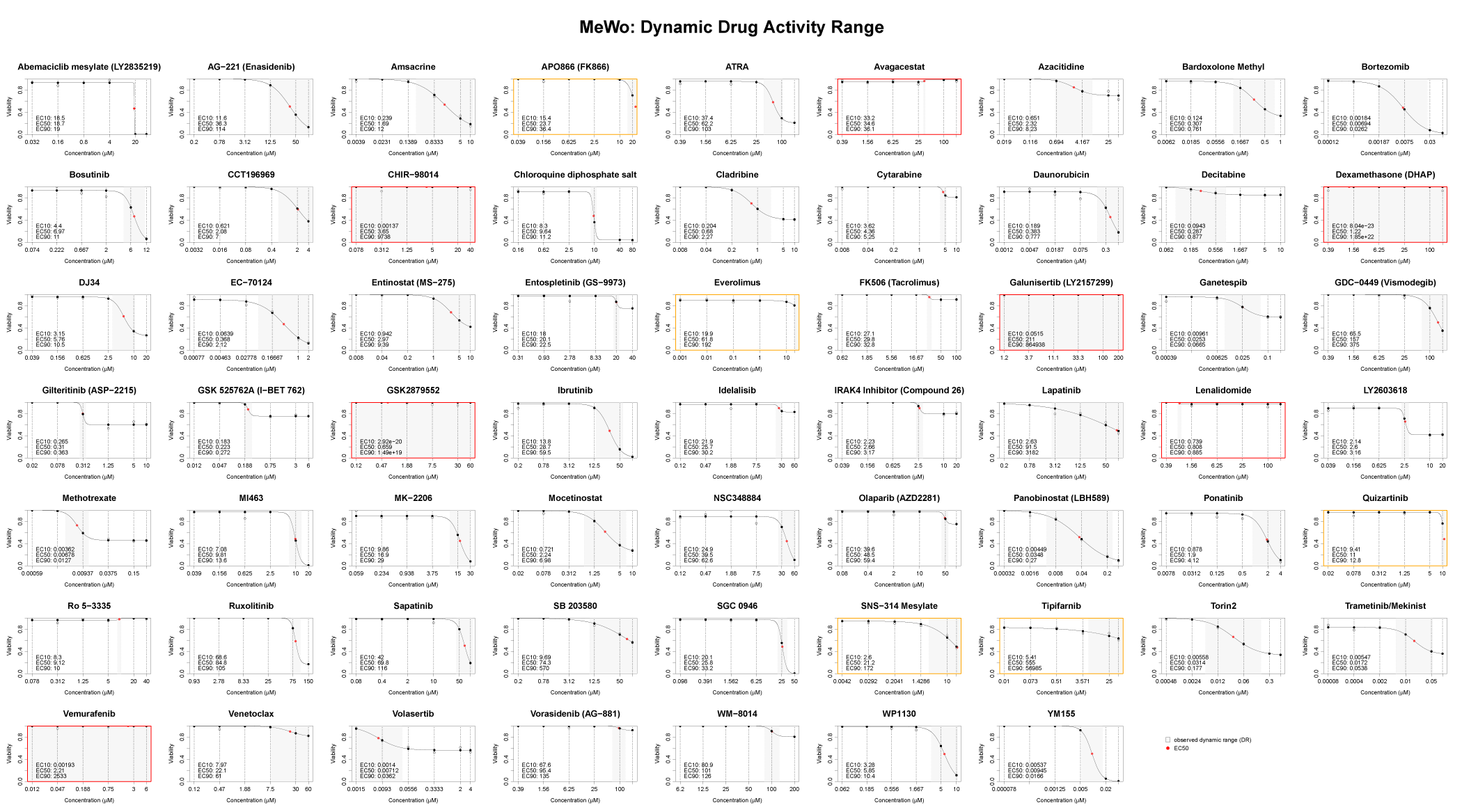


**Supplementary Figure S13.** Dynamic drug-activity range for all drugs. screenwerk highlights inactive drugs or drugs with activity outside the dose range with a red box around the graph.

The average DDAR for a given drug for all cell lines in a given screen can also be plotted, either for individual drugs (Suppl. Fig. S14) or for all the drugs in the screen either in single graph (Suppl. Fig. S15).


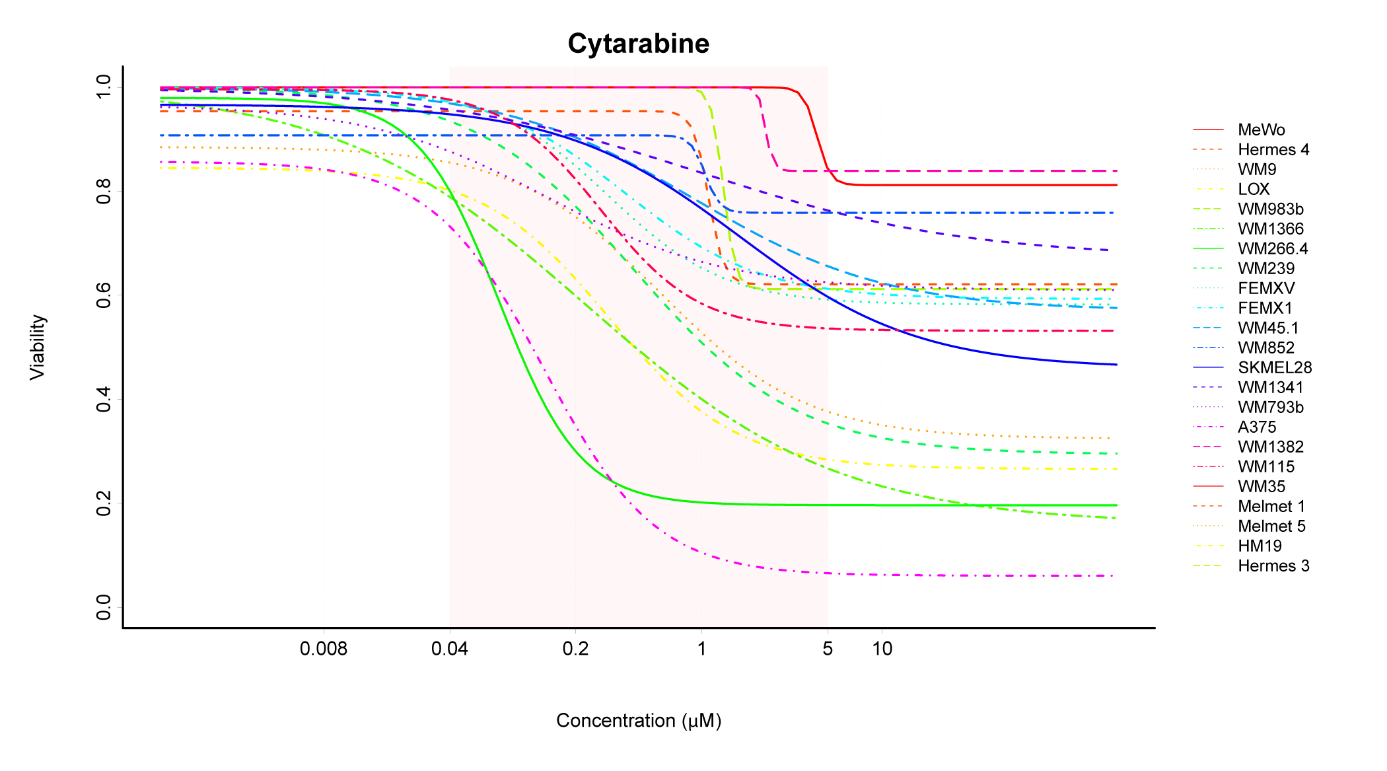


**Supplementary Figure S14.** Dynamic drug-activity range for of a given drug (Cytarabine) for all cell lines in the screen.


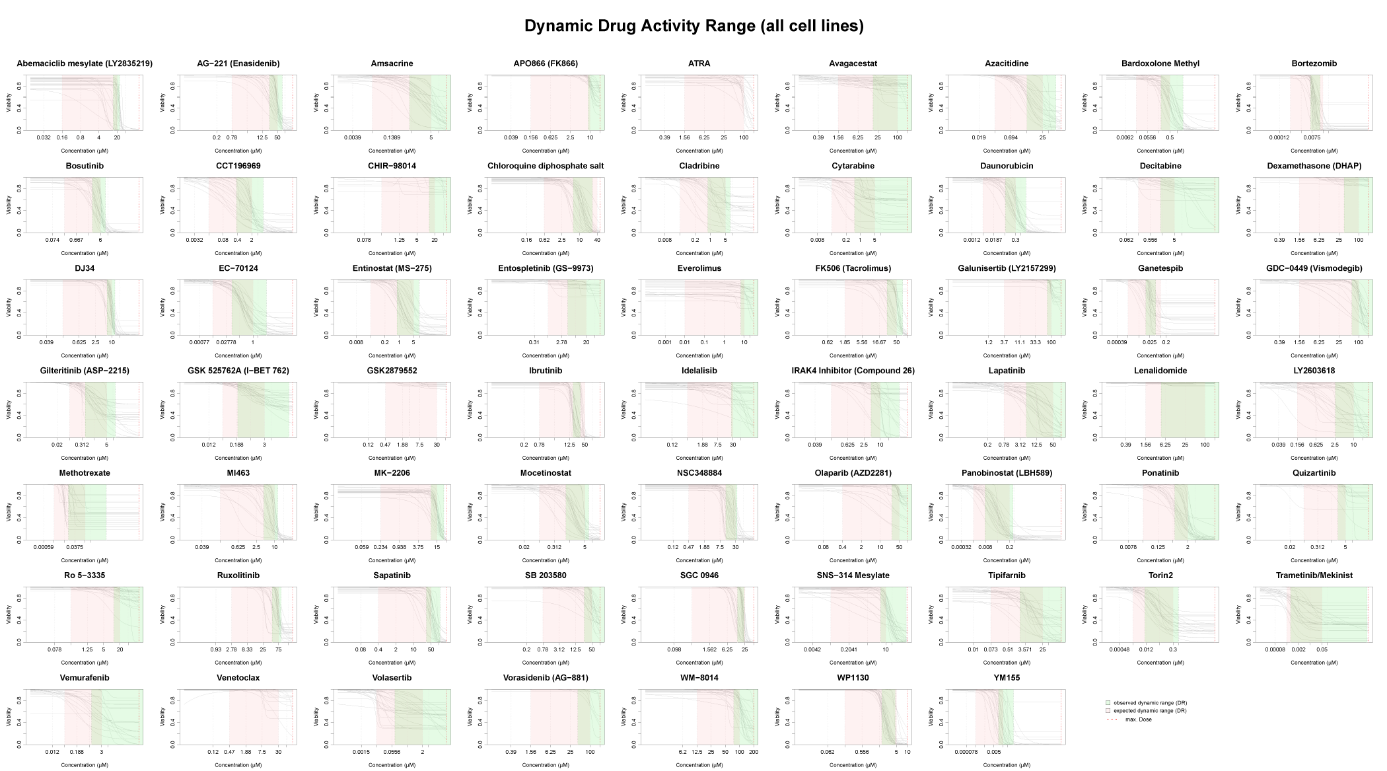


**Supplementary Figure S15.** Dynamic drug-activity range for all drugs and cell lines.

If desired, screenwerk can plot the same data but without indicating the DDAR and without the curves fitted to the points, instead showing the raw fit of the dose responses (Suppl. Fig. S16).


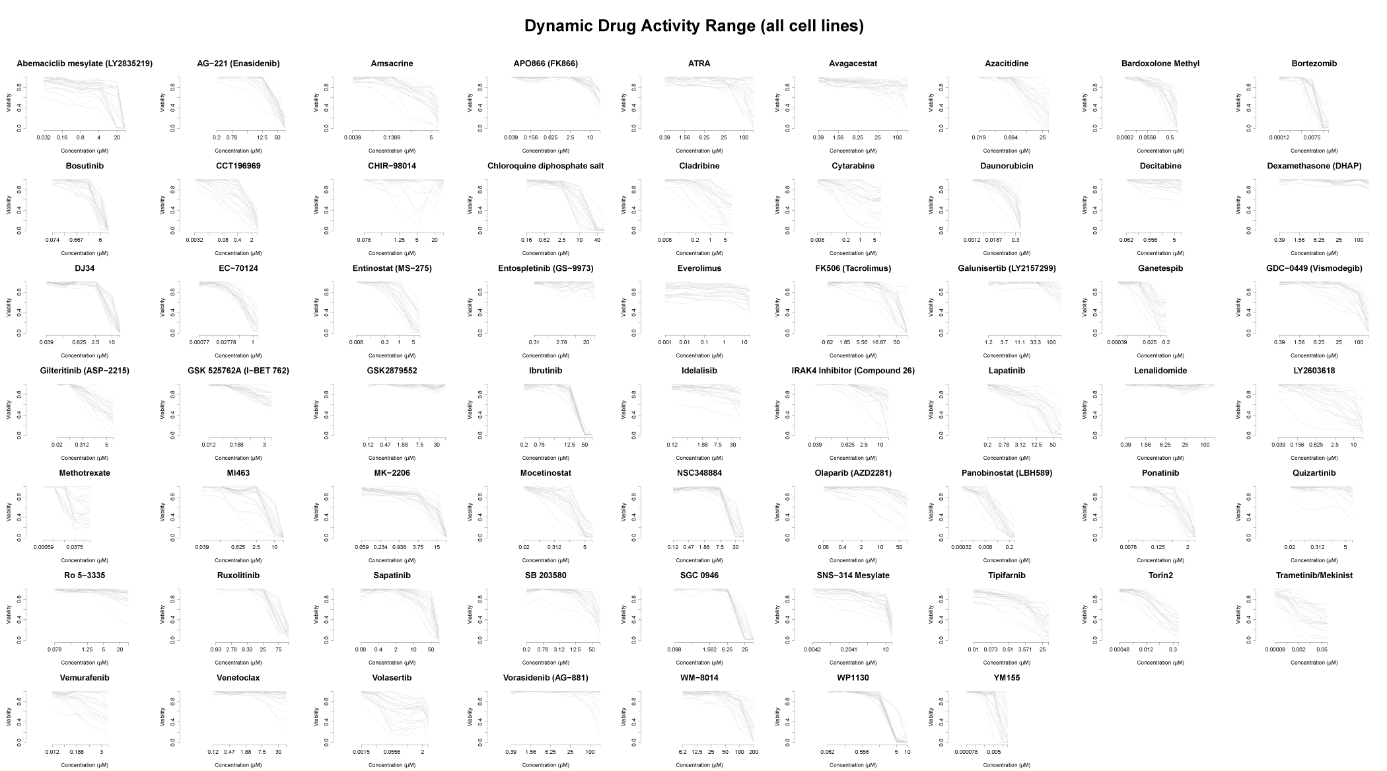


**Supplementary Figure S16.** Unfitted dose response curves for all drugs.

### Synergy analysis with bayesynergy

This particular module is primarily used for the estimation of synergies from the interaction between two drugs across their drug concentration range based on a bayesian semi-parametric model. It calculates synergies using the R-package bayesynergy (Rønneberg *et al.*, 2021). The output of this module is directly streamlined from the output of bayesynergy as a list containing the posterior distribution summaries of the volume under the surface (VUS), which summarize efficacy and interaction effects of the drug combinations, the EC50s based on the monotherapy curves, additional statistics of the fitted bayesyenrgy model and additional quality parameters, including a measure of synergy (bayesfactor). Optionally, the output and bayesynergy plots can be saved to a user specified location.

In the second module linage, the drug combinations are assessed by estimating synergy and antagonism for each individual drug pair. This is being done primarily with the R-package bayesynergy (Rønneberg et al., 2021), which was specifically designed for with high-throughput drug combination screens in mind.

**bayesynergy**(processedData, .saveoutput = TRUE, .plot = TRUE, .saveto)

The function uses data of class S3:processedData and runs bayesynergy with a predefined set of parameters for ease of use. The actual output and plots of bayesynergy can be saved to file. The plots that are generated include both the single-drug responses as well as the responses of the corresponding drug combinations, in addition to a histogram with drug sensitivity scores (DSS) (Suppl. Fig. S17). Furthermore, the non-interaction, interaction and response surface are plotted, as well as the estimated drug combination scores.


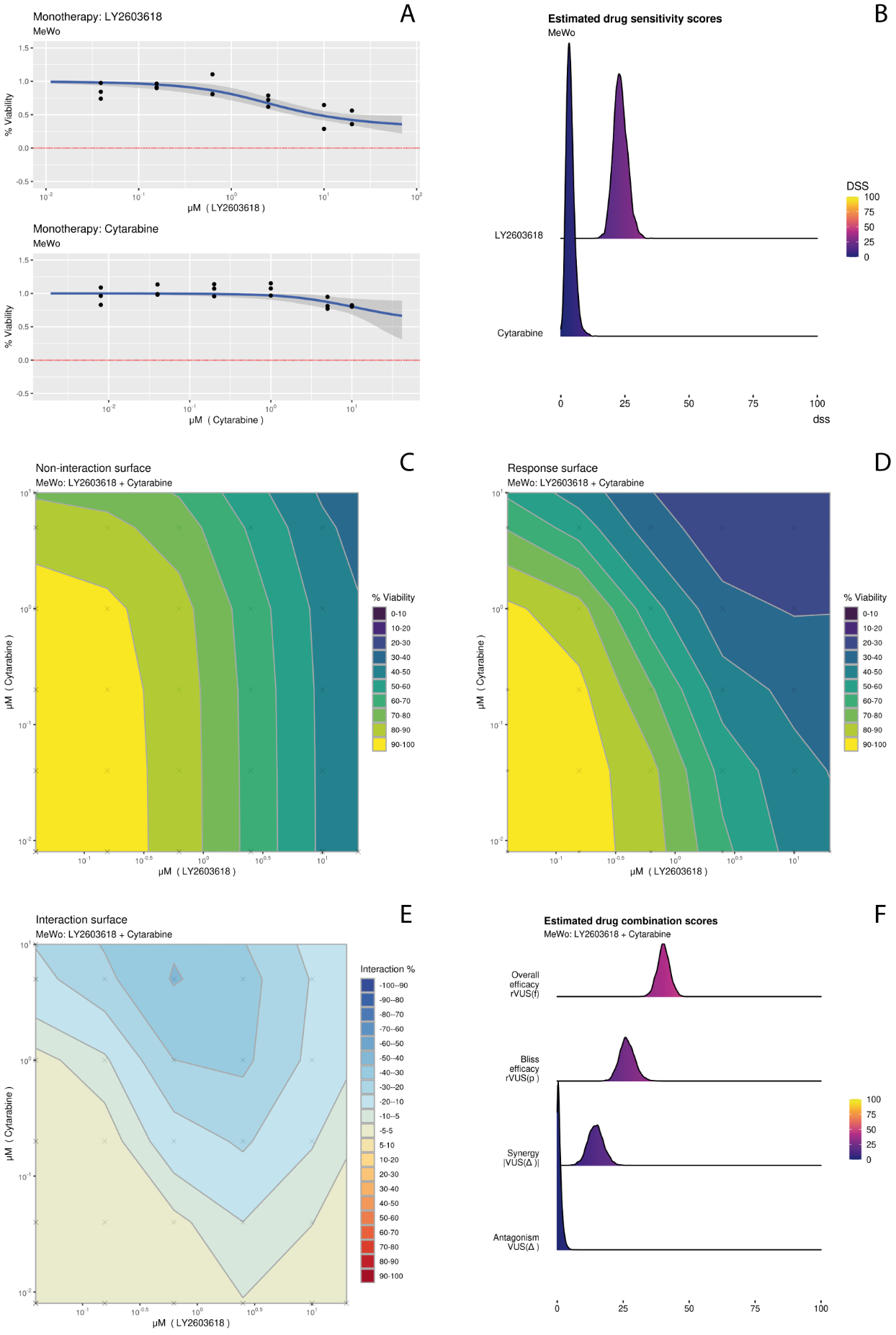


**Supplementary Figure S17. Graphs from bayesynergy**. ***A***, Single drug responses (monotherapies). ***B***, drug sensitivity scores (DSS). ***C***, Example of a non-interaction surface. ***D***, Example of a synergistic response surface. ***E***, Example of a drug interaction surface. ***F***, Example of drug combination scores based on the volume under the surface (VUS) for both a synergistic and an antagonistic combination.

The analytical module for synergy estimation will be extended with other tools over time. One alternative tool already implemented is SynergyFinder (Ianevski *et al.*, 2017), which allows users to use a variety of different synergy scoring methods (Tang *et al.*, 2015; Yadav *et al.*, 2015), such as HSA, Loewe and ZIP. bayesynergy (Rønneberg *et al.*, 2021) was implemented as the principal tool for estimating synergies, due to its ability to account for uncertainty, and to analyze high-throughput drug screens with multiple drug combinations.

### Synergy analysis with synergyfinder

The availability of synergyfinder (Ianevski *et al.*, 2017) extends the function of screenwerk for users who wish to estimate synergies using more traditional and established synergy models

**synergyfinder**(processedData, synergymodel = c("ZIP", "HSA", "Bliss", "Loewe"), .saveoutput, .plot, .saveto)

The function uses data of class S3:processedData and runs synergyfinder with a predefined set of parameters for ease of use. In addition, the output from synergyfinder can be saved to file, as well as a set of plots. Another S3 object is returned with the input data, the synergyfinder output, as well as a data set with the ranked synergy scores based on the synergy model of choice.

### Synergy scoring

With this module the synergies are ranked based on the median synergistic VUS divided by the mean absolute deviation. The antagonistic score is derived from the antagonistic VUS. The synergy score is not the product of the mean synergy and antagonism, but rather decoupled from the antagonism. Even though the primary focus of drug combination screens is usually to find synergies, the importance of the antagonistic response of a drug combination should not be overlooked, since it can be biologically insightful and might have clinical relevance. In cases where the estimation is accompanied with a high uncertainty in its prediction, the corresponding drug combination and synergy scores are flagged yet still retained. The ranked synergy scores can be saved together with the antagonistic scores and several other statistical information along with a number of quality control parameters as a csv file.

The synergy scores can furthermore be visualized in a number of different plots:

**synergyScoring**(bayDFS, .saveoutput = TRUE, .plot = TRUE, .saveto)

The estimated synergies are subject to scoring, in which the drug pairs are ranked based on the highest synergy. In addition to the synergy scores, additional statistical data is being provided along with a number of QC parameters. The data is saved as a csv file and a number of plots can be generated, such as a graph showing the synergy and antagonism scores for all drug pairs (Fig. 1C), the synergy scores for each drug (Suppl. Fig. S18), and a more detailed distribution of synergy scores for a given individual drug in a particular cell line (Suppl. Fig. S19).


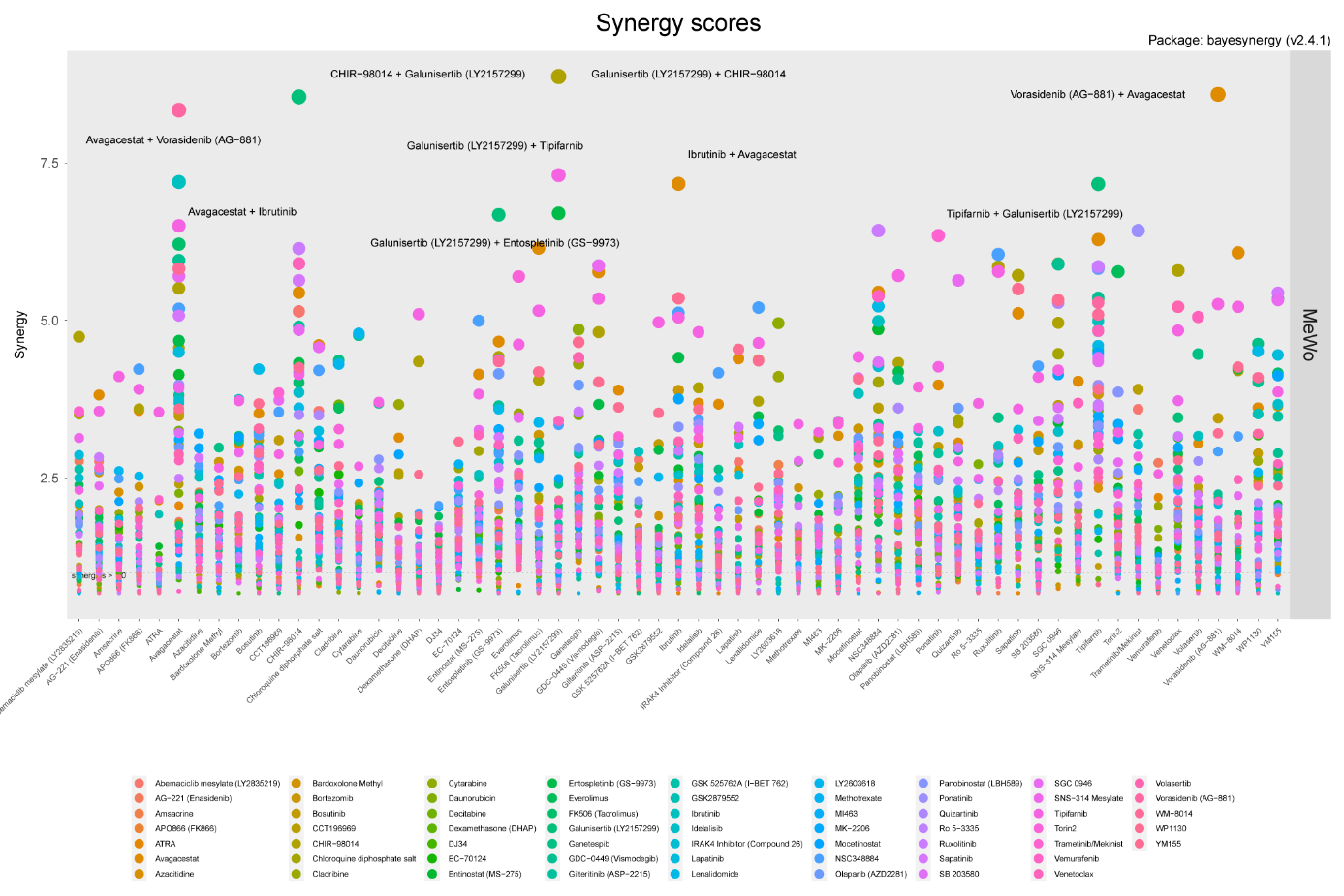


**Supplementary Figure S18.** Drug synergy scores for each drug.


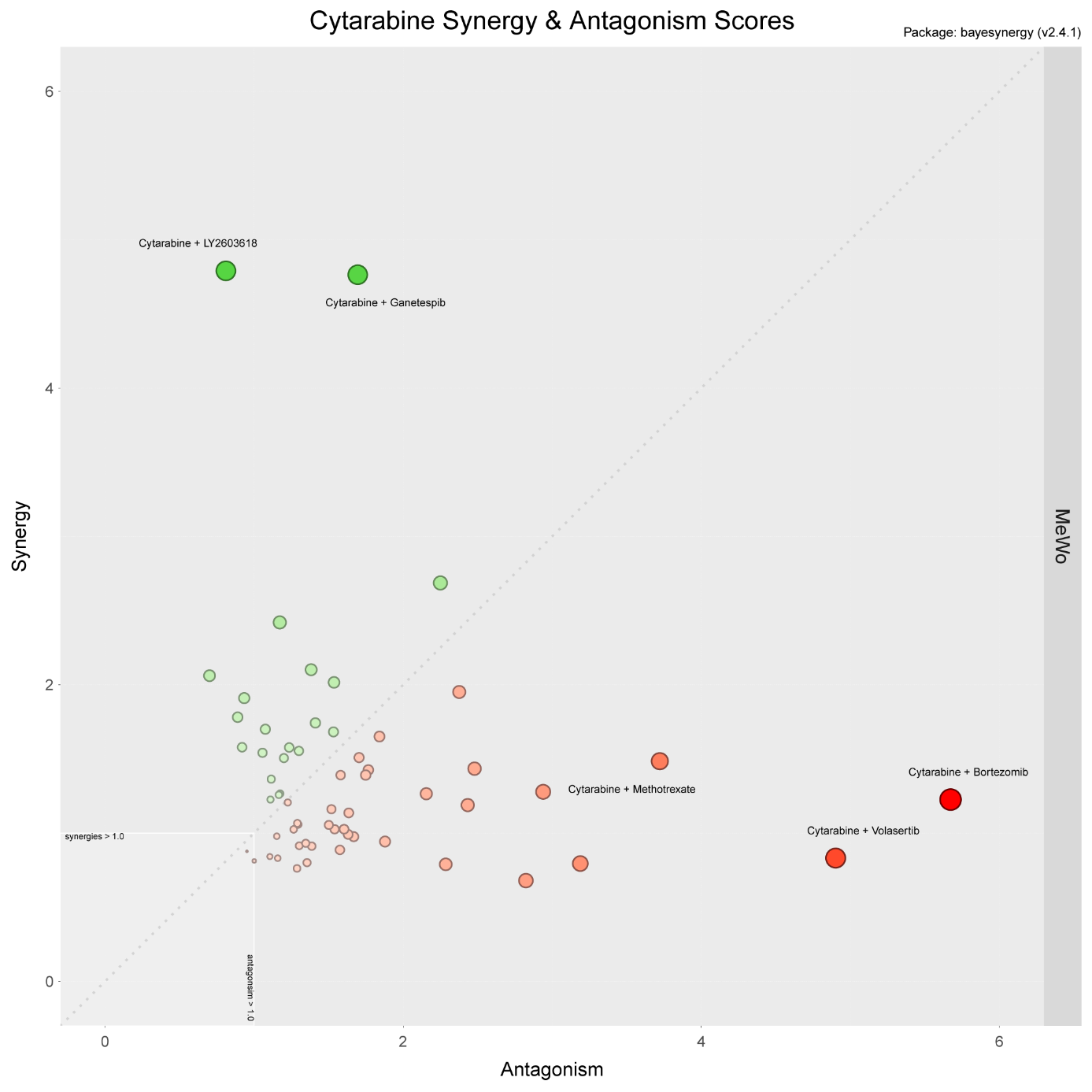


**Supplementary Figure S19.** Drug synergy and antagonism scores for a given individual drug (in this case Cytarabine in MeWo cells).

## Installation

screenwerk can be installed from a github repository using the R-package *devtools*.

install.packages('devtools')

library(devtools)

# Install R-package from github

devtools::install_github('Enserink-lab/screenwerk', build = TRUE, build_opts = c("--no-resave-data", "--no-build-vignettes"))

# Load 'screenwerk' package

library(screenwerk)

## Prerequisite

In order to install and run screenwerk, the following dependencies need to be installed prior the installation of screenwerk: bayesynergy: <https://github.com/ocbe-uio/bayesynergy/>

## Discussion

screenwerk makes it possible to adapt the analysis with a customizable workflow on the basis of its modularity. While coupling multiple individual modules, screenwerk has a strong emphasis on retaining data integrity throughout the pipeline to ensure data quality and consistency. In order to maintain data integrity, screenwerk carries through the consolidation of data from different sources, and performs cleaning and formatting of data into various pre-set formats for different downstream process that are dependent on different data formats. screenwerk necessitates the predefinition of standards and requirements for experimental quality, which significantly reduces variability and increases reproducibility. The quality of the data has to be ensured prior to any analytical proceedings. screenwerk offers an individual module for examining data quality, providing an analytical and visual feedback prior to the analysis.

The screenwerk package was also designed to adapt to technological advances with the ability to up-scale an experimental design whenever necessary. However, up-scaling leads to substantial practical and technical challenges, one of them being the processing of big data and the associated computational challenges. The increase in throughput leading to lower volumes leading to an increase in susceptibility to experimental and technical variation, making it a challenge to separate the signal from the noise of technical and biological variation. With this in mind, screenwerk was developed with a strong focus on quality assurance and data integrity.

screenwerk was also designed to accommodate data integration from various sources, considering that at various stages of the experiment, data from different machines and devices will be generated, all having different formats. Another important objective in mind was the visualization of data throughout the analytical pipeline. At any essential stage of the analysis, data can be reported back in a visual context for easier interpretation of the results and better decision-making for consecutive experiments. screenwerk has profound data analytics and allows the exploration of data by means of visual representation. screenwerk's potential can be integrated throughout different stages of an experiment, from the initiation and design of an experiment, to fundamental stages of data acquisition and processing, to experimental analysis, as well as post-experimental evaluation.

screenwerk guides the user through various stages by offering a number of tools to get started. It offers tools to convert different data formats, import data from different sources, generate machine readable data files, such as dispensing files, that are crucial for the execution of the experiment, design the layout of the experiment, test and validate the experimental design prior the run of an experiment. Another important feature of screenwerk is that it offers features that allow to customize the design of an experiment by choosing a number of different plate formats, allowing to up- or downscale an experiment, work with a wide range of drugs, volumes and drug concentrations, how many replicates to use, the use of different experimental controls, the possibility of randomization and exclusion of wells and much more.

Finally, the output of screenwerk can be used to connect to a variety of modules for the post-experimental phase. It is able to read raw data from selective sources and consolidate different datasets prior any normalization and analytical processing. The analytical modules are separated in modeling the dose response of single drug treatments and the combinatory drug effects, respectively. Both modules provide feedback on the overall quality of the modeling and offer the visualization of a vast amount of data in a representative and meaningful way.

References

Ianevski, A. *et al.* (2017) SynergyFinder. A web application for analyzing drug combination dose-response matrix data. *Bioinformatics (Oxford, England)*, **33**, 2413–2415.

Potdar, S. *et al.* (2020) Breeze. An integrated quality control and data analysis application for high-throughput drug screening. *Bioinformatics (Oxford, England)*, **36**, 3602–3604.

Ritz, C. *et al.* (2015) Dose-Response Analysis Using R. *PloS one*, **10**, e0146021.

Rønneberg, L. *et al.* (2021) bayesynergy. Flexible Bayesian modelling of synergistic interaction effects in in vitro drug combination experiments. *Briefings in bioinformatics*, **22**, bbab251.

Society for Laboratory Automation and Screening ANSI/SLAS Microplate Standards, R2012, no. ANSI SLAS 4-2004. https://www.slas.org/education/ansi-slas-microplate-standards/.

Tang, J. *et al.* (2015) What is synergy? The Saariselkä agreement revisited. *Frontiers in pharmacology*, **6**, 181.

Yadav, B. *et al.* (2015) Searching for Drug Synergy in Complex Dose-Response Landscapes Using an Interaction Potency Model. *Computational and structural biotechnology journal*, **13**, 504–513.

Zhang *et al.* (1999) A Simple Statistical Parameter for Use in Evaluation and Validation of High Throughput Screening Assays. *Journal of biomolecular screening*, **4**, 67–73.
